# Differentiation and Maturation of Muscle and Fat Cells in Cultivated Seafood: Lessons from Developmental Biology

**DOI:** 10.1007/s10126-022-10174-4

**Published:** 2022-11-14

**Authors:** Claire Bomkamp, Lisa Musgrove, Diana M. C. Marques, Gonçalo F. Fernando, Frederico C. Ferreira, Elizabeth A. Specht

**Affiliations:** 1 Department of Science & Technology, The Good Food Institute, Washington, DC USA; 2 University of the Sunshine Coast, Sippy Downs, Queensland Australia; 3 Department of Bioengineering and Institute for Bioengineering and Biosciences, Instituto Superior Técnico, Universidade de Lisboa, Av. Rovisco Pais, 1049-001 Lisbon, Portugal; 4 Associate Laboratory i4HB—Institute for Health and Bioeconomy, Instituto Superior Técnico, Universidade de Lisboa, Av. Rovisco Pais, 1049-001 Lisbon, Portugal

**Keywords:** Cultivated seafood, Cell-based seafood, Signaling, Differentiation, Fat, Muscle

## Abstract

Cultivated meat, also known as cultured or cell-based meat, is meat produced directly from cultured animal cells rather than from a whole animal. Cultivated meat and seafood have been proposed as a means of mitigating the substantial harms associated with current production methods, including damage to the environment, antibiotic resistance, food security challenges, poor animal welfare, and—in the case of seafood—overfishing and ecological damage associated with fishing and aquaculture. Because biomedical tissue engineering research, from which cultivated meat draws a great deal of inspiration, has thus far been conducted almost exclusively in mammals, cultivated seafood suffers from a lack of established protocols for producing complex tissues in vitro. At the same time, fish such as the zebrafish *Danio rerio* have been widely used as model organisms in developmental biology. Therefore, many of the mechanisms and signaling pathways involved in the formation of muscle, fat, and other relevant tissue are relatively well understood for this species. The same processes are understood to a lesser degree in aquatic invertebrates. This review discusses the differentiation and maturation of meat-relevant cell types in aquatic species and makes recommendations for future research aimed at recapitulating these processes to produce cultivated fish and shellfish.

## Introduction

Large-scale industrial meat production causes negative externalities related to the environment, food security, antibiotic resistance, and animal welfare (Godfray et al. 2018; Hilborn et al. 2018). The idea that meat might be cultivated from isolated stem cells has been proposed as a solution to these challenges (Datar and Betti 2010). The concept of cultivated meat (CM) production has been reported in manuscripts published in as early as the 1930s (Birkenhead 1930), while the first patent describing a process to produce meat from cells at large scale was granted in 1999 to Dutch researcher Willem van Eelen (van Eelen et al. 1999). By 2013, the first CM prototype—a beef hamburger—was eaten in a live tasting, and the process of obtaining muscle fibers that composed the cultivated hamburger was further described in a publication the following year (Post 2014). Over the past decade, CM has grown from an idea into a nascent field consisting of various academic labs and for-profit companies (Choudhury et al. 2020; Nyika et al. 2021).

While the primary focus has been on farmed terrestrial animals, companies and researchers are also attempting to cultivate seafood (Rubio et al. 2019a; Tsuruwaka and Shimada 2022; Goswami et al. 2022a). Indeed, one of the first academic publications on CM described an attempt to expand a filet of goldfish meat in vitro as a protein source for long-term space travel (Benjaminson et al. 2002). Although seafood’s impacts vary widely across species, regions, and production practices, both wild-caught fish and aquaculture may be associated with significant challenges (Pauly and Zeller 2016; Boone Kauffman et al. 2017; Watts et al. 2017; Lima et al. 2018; Parker et al. 2018). Cultivated seafood (CS), obtained following cellular agriculture approaches, has the potential to ameliorate many of the negative externalities associated with seafood production (Reis et al. 2021). However, realizing these benefits depends on several discrete, intermediate successes, including the need for CS products to be both cost-competitive and viewed as acceptable substitutes by consumers (Halpern et al. 2021). As of the end of 2021, twenty companies globally were working on CS, with nine of them being established earlier in that year (Azoff 2022).

The relationship between the biology of tissue and the organoleptic and nutritional properties of meat is complex (Listrat et al. 2016). To produce CM, stem cells must be differentiated into mature myofibers, adipocytes, and other meat-relevant cell types (Lee et al. 2022). Therefore, methods for inducing differentiation and maturation that are efficient, cost-effective, and food-safe must be identified. Existing knowledge of cell differentiation pathways should be used to generate hypotheses and identify candidate strategies to promote cells’ differentiation, which may then be empirically tested and optimized. While this knowledge is limited for many seafood species, cellular differentiation pathways are reasonably well understood in zebrafish (Guyon et al. 2007; Salmerón 2018; Keenan and Currie 2019). Indeed, it has been suggested that the wealth of biological information available for zebrafish makes it a good candidate for early studies and perhaps even product development for CS (Potter et al. 2020).

This review discusses molecular signals involved in differentiating muscle, fat, and other cell types necessary for producing high-quality CS. Where possible, data from popular seafood species are discussed. While the primary focus is on fish, which have been better studied, relevant differentiation pathways in aquatic invertebrates are briefly discussed. Where possible, recommendations are provided as to how existing data may be used to inform future efforts to improve differentiation protocols for CS.

## Cell Sources for CS

**Fig. 1:**
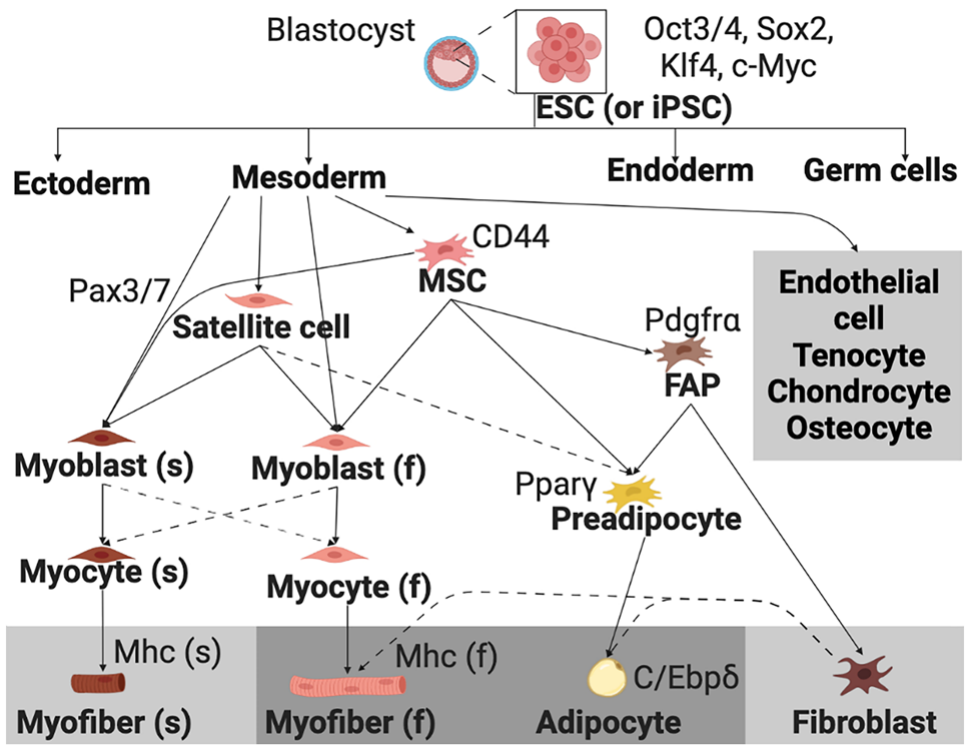
Lineage relationships between cell types that may be used as starting materials for CM and CS and desired cell types likely to be present in the final product (gray background). CS products will be composed primarily of fast myofibers and adipocytes, likely with smaller quantities of some of the other listed cell types. Dashed lines indicate cell type transitions that are not canonically understood to be part of normal developmental or regenerative processes, but that have been observed experimentally (Blagden et al. 1997; Du et al. 1997; Asakura et al. 2001; Potthoff et al. 2007; Tsuruwaka and Shimada 2022) and may be useful for producing the cell types desired in CS products. Some examples of genes/proteins that serve as useful markers are listed next to the associated cell type (Devoto et al. 1996; Todorcević et al. 2010; Bricard et al. 2014; Landemaine et al. 2014; Ma et al. 2018; Peng et al. 2019; Li et al. 2020; Reiss et al. 2021)

Various pluripotent and adult stem cell types have been investigated or proposed as sources for CM and CS production (Reiss et al. 2021). Most of the mature cell types likely to be necessary for CM and CS belong to the mesodermal lineage (Fig. 1 and Table 1). Because the most critical cells for the final product are muscle and fat cells, the precursors to these cell types—mesenchymal stem cells, satellite cells, fibro-adipogenic progenitors, and preadipocytes—are the most likely candidates among the adult stem cells to be used for CM and CS production (Reiss et al. 2021). It was recently demonstrated that fibroblast-like cells isolated from filefish fins could be easily differentiated into several cell types, including skeletal muscle–like cells and adipocytes (Tsuruwaka and Shimada 2022). If similar results are found for other fish species and under serum-free conditions, fish fin–derived fibroblasts could serve as an alternative cell source with the potential to make cell line development and culture substantially easier. Cell types can be identified experimentally by the expression of marker genes, but these markers are somewhat less well characterized in seafood species than amniotes. Methods for isolation, identification, culture, and enhancement of proliferation for some key cell types are discussed below, emphasizing data from fish where possible.

**Table 1:** Advantages and disadvantages of various cell types as starting material for CM and CS

Cell type	Advantages	Disadvantages
ESC-like	Can become any adult cell type, theoretically unlimited proliferation	Difficulty in obtaining embryonic tissue from non-aquacultured species, lack of established differentiation protocols (Reiss et al. 2021)
iPSC	Can become any adult cell type, theoretically unlimited proliferation	Methods are not well-established in fish besides zebrafish (Rosselló et al. 2013; Peng et al. 2019), lack of established differentiation protocols (Reiss et al. 2021), differentiation efficiency may be a challenge (Löhle et al. 2012)
MSC	Can become a wide variety of mesodermal cell types, including most of those relevant to meat	Limited proliferative capacity unless immortalized
SC	Easy to obtain and culture (Reiss et al. 2021)	Limited proliferative capacity unless immortalized, generally only useful as a cell source for myogenic but not adipogenic cell types (but see Asakura et al. (2001))
FAP	Easy to obtain and culture (Reiss et al. 2021)	Limited proliferative capacity unless immortalized, generally only useful as a cell source for adipogenic (and connective tissue) but not myogenic cell types
Preadipocyte	Easy to obtain and culture (Reiss et al. 2021)	Limited proliferative capacity unless immortalized, generally only useful as a cell source for adipogenic but not myogenic cell types
Fibroblast	Easy to obtain and culture; continuous lines from a variety of fish species are available (Thangaraj et al. 2021; Goswami et al. 2022b)	Limited proliferative capacity unless immortalized, generally understood not to be a precursor to muscle or fat unless transdifferentiated. However, a recent study reported differentiation of a fibroblast-like line from one fish species into a variety of cell types without genetic manipulation (Tsuruwaka and Shimada 2022)

### ESC-Like Cells and iPSCs

Embryonic stem cell (ESC)–like lines have been established from several fish species, including sea bass (Chen et al. 2003a; Buonocore et al. 2006; Parameswaran et al. 2007), catfish (Dash et al. 2010; Barman et al. 2014), sea bream (Béjar et al. 1999; Chen et al. 2003b), tilapia (Fan et al. 2017), turbot (Chen et al. 2005), cod (Holen et al. 2010), rohu carp (Goswami et al. 2012), zebrafish (Collodi et al. 1992; Driever and Rangini 1993; He et al. 2006; Ho et al. 2014), and medaka (Wakamatsu et al. 1994; Hong et al. 1996; Yuan and Hong 2017). In contrast to true ESCs from other species such as mouse (West et al. 2006), it is not clear that any of the described ESC-like lines from fish can give rise to germ cells. However, given the cell types involved, existing ESC-like cells are expected to be sufficient as a cell source for most CS products, perhaps with the exception of roe. Many of these studies reported culturing fish ES-like cells in Dulbecco’s Modified Eagle’s Medium (DMEM) with β-mercaptoethanol, selenium, glutamine, pyruvate, non-essential amino acids, fibroblast growth factor 2 (FGF2), leukemia inhibitory factor (LIF), fetal bovine serum (FBS), fish serum, and fish embryo extract (Hong et al. 1996). Under these conditions, fish ES-like cells generally maintain markers of pluripotency. DMEM supplemented with insulin-like growth factor 2 (IGF-2) maintained pluripotent medaka ES-like cells, though with a reduced growth rate relative to FBS-containing media (Yuan and Hong 2017). Leibovitz’s L-15 with FBS has also been shown to support the growth of ES-like cells from some fish species (Bryson et al. 2006; Parameswaran et al. 2007). ESC-like cells from shrimp have been successfully cultured for up to ten passages, though contamination was a substantial problem and ultimately a continuous line was not successfully developed (Fan and Wang 2002).

Zebrafish fibroblasts have been successfully reprogrammed to induced pluripotent stem cells (iPSCs) using the Yamanaka reprogramming factors Oct3/4, Sox2, Klf4, and c-Myc (Rosselló et al. 2013; Peng et al. 2019). More recently, iPSC-like cells were generated from koi fibroblasts using a chemical reprogramming method (Xu et al. 2022). The fact that fish cells can be reprogrammed has the potential to be used for CS, especially for species from which it is difficult to obtain tissues or embryos. Beyond the simple translation of iPSC reprogramming methods to common seafood species, it will be critical to transition to methods not reliant on antibiotic-inducible systems and, ideally, footprint-free methods (Rao and Malik 2012) to alleviate potential concerns related to genetic modification.

### MSCs

Mesenchymal stromal cells (MSCs) are a potential cell source for CS due to their high self-renewal capacity, high proliferation rate, and multipotency. Isolation of MSCs from a variety of fish tissues, including visceral adipose tissue (gilthead sea bream (Salmerón et al. 2016), rainbow trout (Bou et al. 2017), and Atlantic salmon (Ytteborg et al. 2015)), vertebra bone (gilthead sea bream (Salmerón et al. 2016; Riera-Heredia et al. 2019)), heart (zebrafish (Fathi et al. 2019)), and liver (zebrafish (Fathi et al. 2019)) have been reported. Typically in such studies, tissues are mechanically disrupted and digested using collagenase, and MSCs are cultured in DMEM supplemented with 10% FBS.

Adipose-derived MSCs (AMSCs) have been isolated from human adipose tissue obtained by liposuction and processing of the raw aspirate to obtain the heterogeneous cell population known as the stromal vascular fraction (SVF) (Zuk et al. 2001). AMSCs may be also found within adipose tissue of bovines and other mammals (Mehta et al. 2019). Centrifugation of the collagenase-digested tissue results in three-layered fractions: a top fraction containing floating lipids and mature and lysed adipocytes, followed by an aqueous fraction composed of enzymes and medium, and the SVF, where MSCs and adipogenic progenitors can be found (Mehta et al. 2019). Because the SVF is heterogeneous, fluorescence- or magnetic-activated cell sorting (FACS or MACS) may be used to select a more defined population, as demonstrated by Ishimura et al. (2008) in mouse cells. However, the utility of this method depends on the availability of suitable antibodies, which may be a limitation for many aquatic species.

In culture, fish MSCs can be identified by ability to adhere to tissue culture polystyrene plates, specific cell surface markers, lack of hematopoietic and endothelial markers, morphology, and multi-lineage differentiation capacity. Fathi et al. (2019) showed that MSCs from zebrafish heart and liver presented a fibroblast-like morphology and expressed Nanog, Oct4, and Sox2, common pluripotency markers. These cells were positive for CD44 and CD90 and negative for CD31 and CD34. Moreover, zebrafish MSCs could differentiate into osteocyte, adipocyte, and chondrocyte lineages, representing a distinctive characteristic of MSCs (Dominici et al. 2006; Fathi et al. 2019).

For CS applications, the multi-lineage potential of MSCs could be evaluated to grow different relevant tissues, such as muscle and adipose tissue. Adipogenic differentiation of gilthead sea bream, rainbow trout, and Atlantic salmon MSCs can be induced using a differentiation media containing insulin, 3-isobutyl-1-methylxanthine, dexamethasone, and a lipid mixture (Ytteborg et al. 2015; Salmerón et al. 2016; Bou et al. 2017). Myogenic differentiation has not yet been reported in the literature for fish MSCs. However, appropriate physical, mechanical, and bio/chemical conditions (through culture medium supplementation) could induce myoblast formation from mammalian MSCs (Wang et al. 2012a; Xu et al. 2015; Witt et al. 2017), including AMSCs (Zuk et al. 2001, 2002; Zheng et al. 2006).

### Satellite Cells and Myoblasts

Satellite cells (SCs, also called myosatellite cells) (Mauro 1961) are another likely cell source for CM (Hanga et al. 2020). While their canonical role is in muscle regeneration, mouse SCs can be differentiated into myogenic, osteogenic, and adipogenic lineages (Asakura et al. 2001). Hollway et al. (2007) identified a population of presumptive SCs in adult zebrafish originating from the anterior somite. This population contributes to the repair of injured muscle (Seger et al. 2011; Knappe et al. 2015) via asymmetric cell division (Gurevich et al. 2016). SCs in adult zebrafish are concentrated primarily in slow muscle, express the transcription factor paired box protein 7 (Pax7), are characterized by dense heterochromatin, and are located between the muscle cell membrane and the basal lamina (Berberoglu et al. 2017). Pax3 and Pax7 are consistent markers of adult muscle SCs and embryonic myogenic progenitors in species including mice (Asakura et al. 2001; Relaix et al. 2005), chicken, quail (Gros et al. 2005), zebrafish (Yin et al. 2018; Ganassi et al. 2018), pearlfish (Marschallinger et al. 2009), and rainbow trout (Bricard et al. 2014; Villasante et al. 2016), although in fish they may remain briefly expressed during early stages of differentiation (Devoto et al. 2006; Marschallinger et al. 2009; Seger et al. 2011). Other commonly used SC markers were reviewed by Siegel et al. (2013).

Montserrat et al. (2007, 2012) described methods for primary SC culture from gilthead seabream, and similar protocols have been employed for rainbow trout (Fauconneau and Paboeuf 2000; Castillo et al. 2002, 2004). Proportions of SCs in carp decrease with age (Koumans et al. 1991), suggesting that isolation from younger fish is preferable. More recently, a spontaneously immortalized myogenic mackerel cell line has been described (Saad et al. 2022). Culture of myogenic cells from aquatic invertebrates has been even less thoroughly studied, though a recent description of methods for primary culture of lobster myogenic cells (Jang et al. 2022) may pave the way for future studies.

Myostatin, a transforming growth factor β (TGF-β) superfamily member, is well-characterized as an inhibitor of proliferation and differentiation in mammalian muscle. Loss of function of myostatin also increases muscle growth in many fish species (Lee et al. 2009; Chisada et al. 2011; Gao et al. 2016; Torres-Velarde et al. 2020), though not in all fish species tested (Terova et al. 2013). Evidence from mammals (Morissette et al. 2009) and fish (Liu et al. 2020) indicates that myostatin inhibits Akt, a positive regulator of muscle hypertrophy. In partial contrast to mammalian systems, myostatin inhibited proliferation but not differentiation in rainbow trout SCs (Seiliez et al. 2012; Garikipati and Rodgers 2012a, b). Also unlike in mammals, it has been suggested that the effects of fish myostatin are not specific to muscle (Gabillard et al. 2013). Myostatin loss of function, therefore, offers a possible strategy for improving proliferation rates as part of the CS bioprocess, but this strategy will need to be tested and may be effective in only some species.

Whereas IGFs in mammalian muscle tend to promote differentiation (Retamales et al. 2015; Pourquié et al. 2018), their effects in fish may be more complex. IGF-1 and IGF-2 stimulated proliferation in primary cultures of myoblasts or SCs from rainbow trout (Castillo et al. 2004; Gabillard et al. 2010; Garikipati and Rodgers 2012a, b). In cultured gilthead seabream SCs, treatment with IGF-2 and IGF-1 induced markers of early and later stages of differentiation, respectively (Jiménez-Amilburu et al. 2013). Consistent with this, IGF-2 was more effective than IGF-1 in stimulating myoblast proliferation (Rius-Francino et al. 2011). Together, these results suggest that IGF-2 and IGF-1 may tend to promote proliferation and differentiation, respectively, but that their effects depend on species and culture conditions. IGF-1’s effects on rat myoblast differentiation are partially mediated through myostatin (Retamales et al. 2015), which lacks its canonical function as an inhibitor of differentiation in at least some fish species. Treatment with specific IGFs—or IGF mimics—during the proliferation or differentiation stages may be helpful for CS, but predicting their effects is not entirely straightforward.

The adenylate cyclase activator forskolin promoted proliferation without effects on differentiation in both zebrafish and mouse SCs (Xu et al. 2013). Anthocyanidin treatment of rainbow trout primary myogenic cells increased expression of Pax7 and non-significantly reduced expression of differentiation markers (Villasante et al. 2016), suggesting that these compounds might help maintain myogenic stem cells in a proliferative state.

### Fibro-/Adipogenic Progenitors (FAPs)

Fibro-/adipogenic progenitors (FAPs) are multipotent non-myogenic MSCs that can be isolated from the muscle’s SVF (Joe et al. 2010; Low et al. 2017). FAPs can be found in the interstitial space of skeletal muscle and support myogenic development and regeneration following muscle injury (Joe et al. 2010). Low et al. described protocols to isolate FAPs from the SVF of murine skeletal muscle using antibodies to cell-specific surface antigens and FACS, resulting in adhered cells with a spindle shape with short projections (Low et al. 2017).

FAPs express the platelet-derived growth factor receptor-α (PDGFRα or CD140a) but can be also recognized by the expression of vimentin, delta like non-canonical Notch ligand 1 (Dlk1) or preadipocyte factor 1 (Pref1), and stem cells antigen 1 (Sca1) (Li et al. 2020). Additionally, in mice, such cells are identified by the absence of CD31, CD45, and integrin-α7 (Uezumi et al. 2011; Judson et al. 2017).

Different studies using mammalian cells, including bovines, had shown the potential of FAPs to differentiate into fibroblasts (Joe et al. 2010) and adipocytes (Arrighi et al. 2015; Uezumi et al. 2010). Therefore, FAPs have been proposed as a cell source for CM (Melzener et al. 2021; Dohmen et al. 2022), namely, for production of connective and fat tissues of meat (Reiss et al. 2021). It remains to be determined whether an equivalent population exists in fish.

FAP proliferation and determination are highly dependent on the niche environment, regulated by crosstalk with SCs, myotubes, and immune cells (Biferali et al. 2019). While proliferation is positively regulated with interleukin-4 (IL-4), interleukin-15 (IL-15), TGF-β1, and myostatin, the commitment to either adipogenesis or fibrogenesis is more complex (Li et al. 2020). Further studies are required for their elucidation and to understand similarities and differences between mammals and fish. The control of those pathways will be crucial to improve CM and CS production efficiency.

### Preadipocytes

Adipogenesis is characterized by two phases—determination and differentiation—requiring progressive induction of genes responsible for functions such as lipid uptake and the secretion of adipokines. Cells that have undergone determination—and thus are committed to the adipocyte lineage, but are not yet differentiated—are often called preadipocytes (Salmerón 2018).

Studies in mice indicate that adipogenic precursor cells express mesenchymal markers such as SCA1, CD34, and CD29 but do not express mice hematopoietic (CD45) and endothelial (CD31) markers. As mouse adipogenic precursors become further committed to the adipocyte lineage, they lose their expression of CD24 (Berry and Rodeheffer 2013; Hepler et al. 2017). Mouse preadipocytes also express the zinc finger protein ZFP423 (Gupta et al. 2010).

Preadipocytes from Atlantic salmon have a fibroblast-like morphology and do not contain lipid droplets (Vegusdal et al. 2003). Identified preadipocyte markers may differ somewhat between fish species but include *peroxisome proliferator-activated receptor gamma* (*pparγ*); *CCAAT/enhancer-binding protein* (*c/ebp*), namely, *c/ebpα* and *c/ebpβ*, *transgelin*, and *fatty acid synthase* (*fas*) in Atlantic salmon (Todorcević et al. 2010); and *glucose-6-phosphate dehydrogenase* (*g6pdh*) in sea bream (Salmerón et al. 2016; Salmerón 2018).

Like MSCs and FAPs, fish preadipocytes can be isolated directly from the SVF (Todorcević et al. 2010; Wang et al. 2012b; Liu et al. 2018; Salmerón 2018). In zebrafish, pancreatic white adipose tissue starts to develop 12 days after fertilization, followed by an increase in visceral, subcutaneous, and cranial depots (Imrie and Sadler 2010). Gilthead seabream preadipocytes obtained from fish specimens weighing 50 g have improved proliferative capacity compared with the ones sourced from 500 g fish specimens (Salmerón et al. 2013), suggesting that the age of the donor animal is an important factor.

The induction of the Wnt/β-catenin pathway has been shown to maintain preadipocytes in an undifferentiated and proliferative state in mammals (Ross et al. 2000) and in carp (Liu et al. 2018). Supplementing the culture medium with IGF-1, insulin, and the growth hormone somatotropin has been shown to improve the proliferation capacity of preadipocytes from gilthead seabream (Salmerón et al. 2013). Insulin promoted, while tumor necrosis factor alpha (TNFɑ) and docosahexaenoic acid (DHA) inhibited, the proliferation of preadipocytes from large yellow croaker (Wang et al. 2012b). Some of these molecules may offer opportunities for optimization of preadipocyte proliferation media, thereby enabling greater efficiency in CS.

## Myogenesis in Fish

The main transcription factors, structural proteins, morphogenetic processes, and other critical elements of muscle fiber formation and function are conserved across the vertebrate lineage, including in non-teleost fish species such as sturgeon (Steinbacher et al. 2006). However, both Xu et al. (2000) and Costa et al. (2002) found that zebrafish, chick, and mouse begin to express myogenesis-related genes in different orders, suggesting differences in the underlying regulatory network. Differences in myosin composition during the early stages of regeneration suggest that different pools of cells might be involved in zebrafish cells’ myogenesis compared to seabream (Rowlerson et al. 1997). Thus, caution is warranted in generalizing results from amniotes to fish or even between different fish species. Conservation of signaling pathways across species means that findings from one species offer a promising hypothesis for another species, but such hypotheses must be carefully tested and not simply assumed to be true.

### Markers of Myogenic Differentiation

**Fig. 2:**
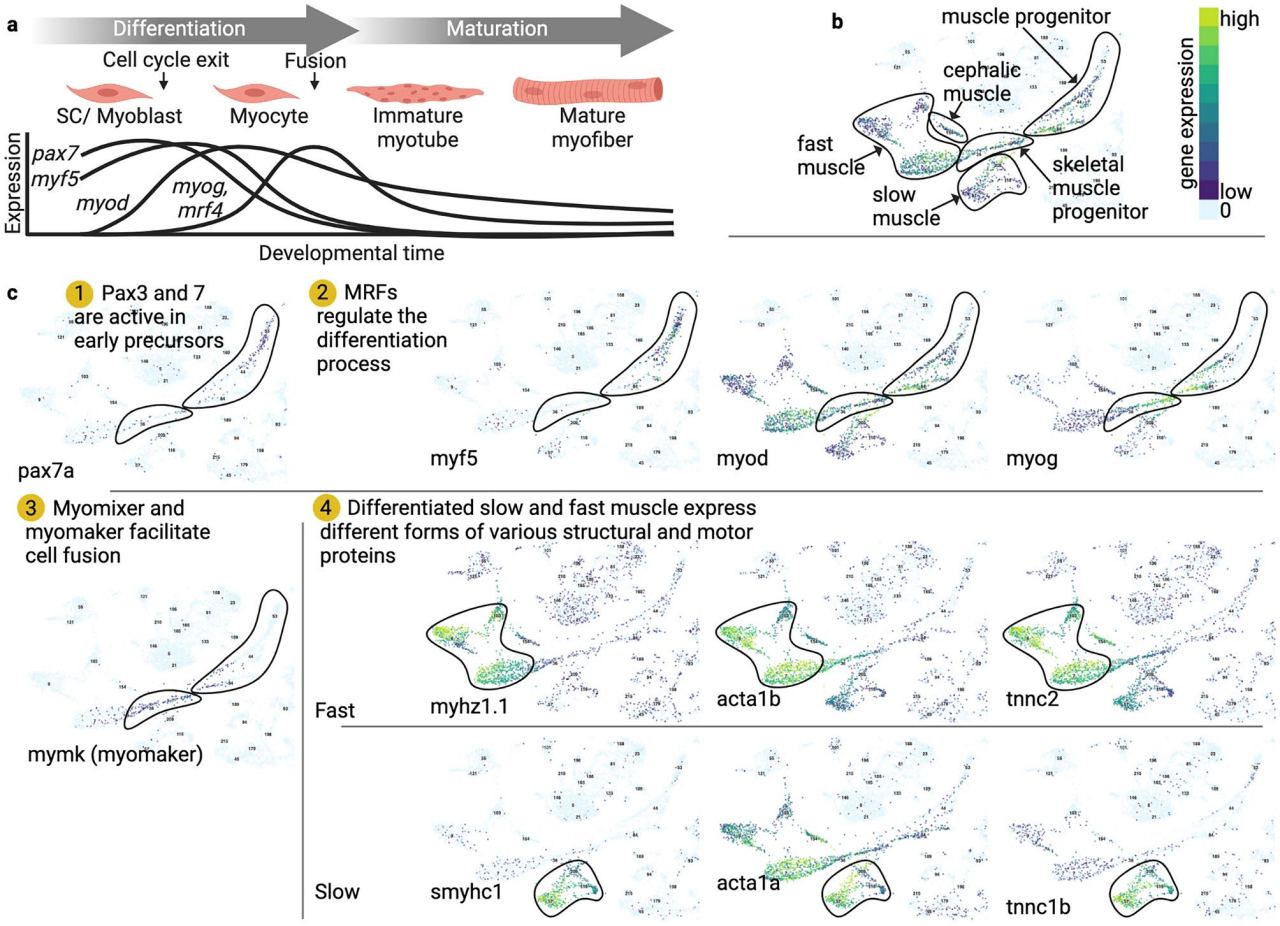
Genes involved in fish muscle differentiation and maturation. **a** Steps involved in the differentiation and maturation process. Line graph schematic shows the approximate timing of activation and downregulation of Pax7 and the four MRFs relative to these steps (Weinberg et al. 1996; Hinits et al. 2007; Schnapp et al. 2009; Chen and Galloway 2014; Farnsworth et al. 2020). **b** UMAP plot of gene expression data (Farnsworth et al. 2020) from developing zebrafish reveals several clusters corresponding to developing and mature muscle. Manually drawn borders correspond to higher level categories, e.g., slow muscle, some of which contain several distinct, numbered clusters. Gene expression data in this panel is from *myod*, shown for reference. Legend applies to panels b and c. Created using the UCSC Cell Browser (https://zebrafish-dev.cells.ucsc.edu) (Speir et al. 2021). **c** Expression of several genes relevant to muscle development or function is shown, with key steps in the development process outlined (Farnsworth et al. 2020; Speir et al. 2021). *Mrf4* (not shown) shows a similar temporal pattern to that of *myog* (Schnapp et al. 2009)

Most of the genes used to mark various stages of myogenesis in fish (Fig. 2) are the same as those used in other vertebrates, though some species differences exist in the expression pattern across cell types or stages.

Myoblast determination protein (MyoD) and myogenic factor 5 (Myf5) mark cells committed to the myogenic lineage in most vertebrate species, including fish (Weinberg et al. 1996), and together with muscle regulatory factor 4 (Mrf4) and myogenin (Myog) are referred to as the muscle regulatory factors (MRFs). In rainbow trout SC cultures, *myoD* expression appears 4–48 h after seeding and remains following differentiation (Rescan et al. 1994). Subsequent studies in zebrafish (Weinberg et al. 1996; Coutelle et al. 2001), pacu (de Almeida et al. 2008), and flounder (Zhang et al. 2006) found high *myoD* transcript levels in somites and juvenile muscle tissue and lower levels in adult muscle. Expression of *myf5* follows a similar pattern but shows a slightly earlier onset of expression and a sharper decline between differentiating and mature muscle tissue (Chen et al. 2001; Coutelle et al. 2001; Farnsworth et al. 2020).

Expression of *myog* in zebrafish cells follows that of *myoD* by several hours and is found in a subset of the *myoD*-expressing cells (Weinberg et al. 1996). Myog expression is commonly used as a marker for the transition from proliferation to differentiation in fish (Millan-Cubillo et al. 2019) and rodents (Asakura et al. 2001).

Xu et al. (2000) found that genes for muscle-specific sarcomeric proteins become expressed at different times throughout zebrafish somitogenesis (see Fig. 2), making them valuable markers of differentiation and maturation stages. Myosin heavy and light chain isoform composition changes as fish mature (Martinez et al. 1991; Veggetti et al. 1993; Johnston et al. 1998; Cole et al. 2004), suggesting that particular myosin isoforms may serve as markers of immature or mature fibers. Proteins such as alpha-actinin, titin, F-actin, and desmin are found in a striated pattern, which may be used to assess the extent of maturation and confirm the proper organization of muscle fibers (Costa et al. 2002; Ganassi et al. 2018).

A variety of markers are commonly used to identify slow muscle fibers and their precursors, including slow myosin heavy chain (Roy et al. 2001; Wolff et al. 2003; Baxendale et al. 2004; Hinits et al. 2009; Yao et al. 2013; Ganassi et al. 2018) and the transcription factors PR domain containing 1 (Prdm1/U-boot) (Hinits et al. 2009; Yin et al. 2018), prospero homeobox 1 (Prox1) (Roy et al. 2001; Wolff et al. 2003; Baxendale et al. 2004; Seger et al. 2011), and myocyte enhancer factor 2ca (Mef2ca) (Hinits et al. 2009). The Sonic hedgehog (Shh) target Patched1 (Ptc1) marks slow muscle precursors (Hinits et al. 2009). Markers of fish fast muscle include fast myosin heavy and light chains (Xu et al. 2000; Hinits et al. 2009; Yao et al. 2013; Ganassi et al. 2018), although *myosin light chain 2 (mylz2)* transcript is also weakly expressed in immature slow muscle (Hinits et al. 2009). Other markers include *muscle α-actin (acta1), α-tropomyosin (tpma), troponin C (tnnc), troponin T (tnnt),* and *parvalbumin (pvalb)* (Xu et al. 2000). In gilthead seabream, *myoD* is found in two isoforms, which may be differentially expressed between slow and fast muscle (Tan and Du 2002).

### Signals Initiating Myogenic Commitment, Fusion, and Maturation

**Fig. 3:**
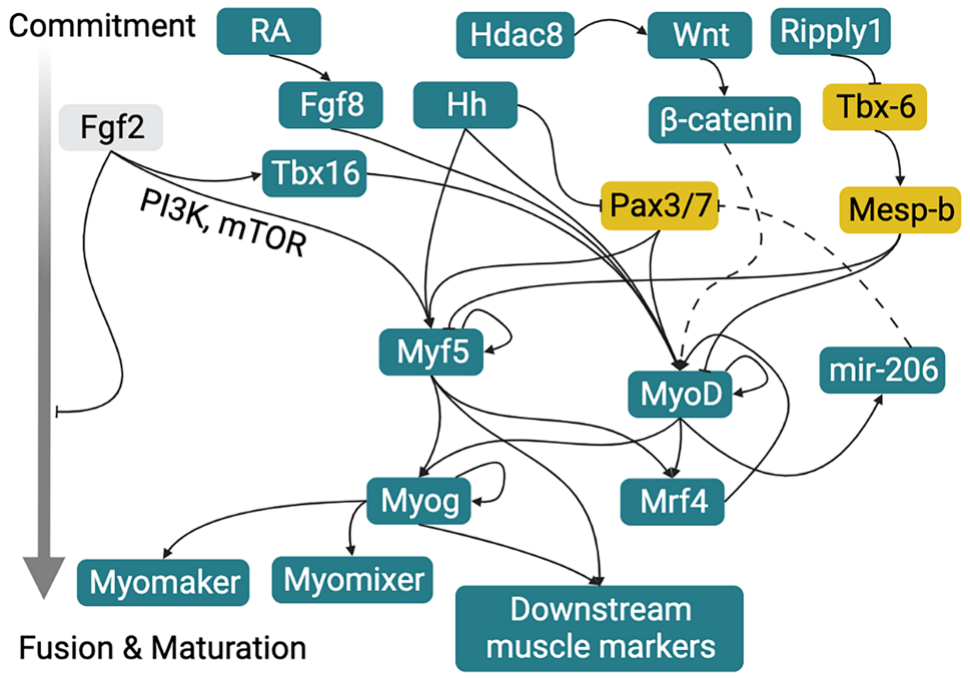
Signaling pathways involved in myogenic commitment, differentiation, fusion, and maturation in fish (Weintraub 1993; Maroto et al. 1997; Hamade et al. 2006; Feng et al. 2006; Hinits et al. 2009, 2011; Schnapp et al. 2009; Xu et al. 2013; Windner et al. 2015; Abraham 2016; Ferrari et al. 2019; Osborn et al. 2020). Gold indicates proteins primarily associated with maintenance of quiescent or proliferative states, teal indicates those primarily associated with differentiation, and gray indicates a mixed role. Dashed lines indicate interactions based on evidence in mammalian systems that have not been directly observed in fish (Kim et al. 2008; Dey et al. 2011)

Various cues control the transition from stem cell to mature myofiber, some of which are outlined in Fig. 3. In cases where differences exist, the differentiation process will be discussed primarily in the context of fast muscle fibers; signals specific to slow fibers are discussed in the “Signals Regulating the Decision to Become Fast or Slow Fibers” section.

#### MRFs

In the absence of both MyoD and Myf5, no muscle is formed in zebrafish embryos (Schnapp et al. 2009; Hinits et al. 2011). Although zebrafish Mrf4 does not usually participate in myogenic commitment (Hinits et al. 2009), ectopic *mrf4* expression in *myoD/myf5* morphant embryos induced expression of MyoD and myogenic commitment (Schnapp et al. 2009). For cultivated fish, inducing expression of *mrf4* alone may therefore be a viable means of inducing both myogenic commitment and differentiation. Besides its role in promoting differentiation, Mrf4 is necessary for proper myofiber alignment in vivo (Wang et al. 2008). Zebrafish MyoD has been suggested to promote myogenesis by inducing the expression of *myog* (Hinits et al. 2009) and miR-206 (Hinits et al. 2011).

#### Fgf

Fgf signaling induces *myod* expression (Groves et al. 2005) via T-box16 (Tbx16) (Osborn et al. 2020). Consistent with this, overactivation of Fgf signaling causes zebrafish embryonic Pax7-expressing cells to differentiate into fast muscle, while inhibition leads to an overabundance of Pax7-expressing cells (Yin et al. 2018). Retinoic acid has been shown to bi-directionally modulate *myoD* and *myog* expression via Fgf8, preferentially in what will become fast fibers (Hamade et al. 2006). While treatment of zebrafish embryonic cells with FGF2 induces *myf5* and *mylz2* expression, expression of those genes was blocked by phosphoinositide 3-kinase (PI3K) or mammalian target of rapamycin (mTOR) inhibitors (Xu et al. 2013). However, FGF treatment also slightly impaired fusion into myotubes (Xu et al. 2013). Fgf promotes dedifferentiation in regenerating zebrafish extraocular muscles (Saera-Vila et al. 2016), and can stimulate the proliferation of committed myoblasts (Gabillard et al. 2010). These latter observations suggest that Fgf in fish may promote myogenic commitment and early differentiation while limiting later stages of differentiation, fusion, and maturation, consistent with findings in other model systems (Moore et al. 1991; Olwin and Rapraeger 1992; Hutson et al. 2010).

#### Wnt Pathway

It has been demonstrated in mouse C2C12 cells that Wnt signaling promotes myogenic differentiation (Abraham 2016), likely via β-catenin’s ability to interact with and enhance the activity of MyoD (Kim et al. 2008). The role of the Wnt pathway in fish myogenesis has been less well studied, though it has been shown that histone deacetylase 8 (Hdac8) positively regulates differentiation via the Wnt pathway (Ferrari et al. 2019).

#### Myomixer and Myomaker

Although the activation of muscle-associated genes and the fusion of myoblasts into multinucleated myofibers occur concurrently in vivo, they can be decoupled experimentally. The membrane-associated peptide Myomixer acts in concert with the transmembrane protein Myomaker to trigger the fusion of mammalian myoblasts, but does not affect myosin expression (Bi et al. 2017). The function of this peptide appears to be conserved across mammalian and fish lineages (Bi et al. 2017). Consistent with the idea that Myomixer and Myomaker specifically affect fusion and not differentiation generally, zebrafish embryos with a Myomaker loss of function show impaired fusion without losing expression of muscle-specific markers (Landemaine et al. 2014; Zhang and Roy 2017). Fusion primarily depends on activation of Myomaker and Myomixer by Myog, though some cells in the medial myotome remain fusion-competent due to activation of Myomaker by notochord-derived Hedgehog (Ganassi et al. 2018). However, it has also been demonstrated that Hedgehog overexpression inhibits expression of Myomixer (Wu et al. 2022) and Myomaker (Shi et al. 2018), leading to defects in fusion. This seeming discrepancy might be explained by the characteristics of different cell populations or by different responses to moderate versus high levels of Hedgehog.

#### Mesp-b, Tbx6, and Ripply1

In zebrafish, mesoderm posterior homolog B (Mesp-b) maintains embryonic muscle progenitors in an undifferentiated, proliferative state by inducing mesenchyme homeobox (*meox1*) expression and inhibiting myoD and *myf5* expression (Windner et al. 2015). Ripply1 causes cells to differentiate by degrading Tbx6, an upstream regulator of *mesp-b* and *ripply1* itself (Kinoshita et al. 2018). Thus, in the context of a cultivated fish bioprocess, manipulating the balance between Mesp-b/Tbx6 and Ripply1 could help maintain myogenic stem cells in an undifferentiated state or induce their differentiation.

#### Alignment, Stiffness, and Other Physical Cues

Myogenic commitment can be induced and maturation enhanced in vitro by various physical cues, including alignment, matrix stiffness, stretch/strain, and electrical stimulation (Lee et al. 2022). Human MSCs could be steered toward the myogenic lineage when grown on micropatterned fibronectin stripes (Yu et al. 2013). Increasing the alignment of myoblasts or MSCs by growing them on decellularized plants (Campuzano et al. 2020; Allan et al. 2021) or curved substrates (Wang et al. 2012a; Connon and Gouveia 2021) also enhances differentiation. Commitment and differentiation can also be influenced by the stiffness of the substrate on which the cells are grown (Engler et al. 2004, 2006; Freeman and Kelly 2017). To our knowledge, the effects of physical cues on myogenic differentiation have not been investigated in fish cells. Determining whether these effects exist in fish—and, if so, what specific values for variables such as stiffness and groove width most efficiently induce myogenesis and how these variables act in combination—may substantially improve product quality and scalability for CS.

### Inducing Myogenesis in Culture

Ultimately, a successful CS bioprocess will require a reliable and efficient means of inducing myogenic commitment, differentiation, and maturation at the desired time. Table 2 summarizes several protocols demonstrated to induce myogenesis in cultured fish cells. Future work should use these results as a starting point, together with a detailed understanding of molecular pathways involved in myogenic differentiation that may be insufficiently activated by the published protocols—as well as other strategies such as the manipulation of cellular alignment—to guide the development of sufficiently robust methods.

**Table 2:** Examples of protocols shown to successfully induce or accelerate myogenic commitment/differentiation in fish cultures

Species and cell type	Treatment	Effect	Citation
Primary trout satellite cells	DMEM + 2% FBS (differentiation medium) Compared to F10 + 10% FBS as control (proliferation medium)	Moderate levels of differentiation (two-fold and 40-fold higher percentage of cells expressing Myog and MyHC, respectively), low levels of proliferation	(Gabillard et al. 2010)
	Proliferation medium supplemented with 50 nM IGF1, 50 nM IGF2, or 0.2 nM FGF2	Five, four, and two-fold increase, respectively, in % of BrdU + cells relative to non-supplemented proliferation medium	
Primary trout satellite cells	DMEM + 10% FBS	Cells differentiate to form large myotubes by day 10 in culture	(Castillo et al. 2002, 2004)
Zebrafish ESC-like cells	1 ng/mL FGF2	Induction of muscle-specific genes including *pax7*, MRFs, *mylz2*	(Xu et al. 2013)
	SB415286 (a GSK3b inhibitor, applied together with FGF2)	Increased expression of *myf5* and *mylz2*, indicating an acceleration in myogenic commitment and differentiation	
	3 GSK3b inhibitors, 2 calpain inhibitors, or the adenylate cyclase activator forskolin (applied individually without FGF2)	Treated cells expressed *myf5* and *mylz2* while controls did not Only forskolin rescued myogenesis in embryos deficient in *fgf4* and *fgf8*	
Zebrafish ESC-like cells	1 ng/mL FGF2 (added at the time of plating)	Treated cells express *myf5* and *mylz2* after 26 h in culture	(Ciarlo and Zon 2016)

Other methods for inducing myogenic differentiation have been demonstrated in cultured mammalian cells (Zhu et al. 2014) but not reported in fish. The reported strategies rely on myogenic inducers such as a specific skeletal muscle cell growth medium (SkGM™)-2 BulletKit™ (Lonza) containing human epidermal growth factor (hEGF), fetuin, FBS, dexamethasone, and insulin, supplemented with 5-azacytidine (Stern-Straeter et al. 2014; Okamura et al. 2018). Messmer et al. (2022) found that transferrin, insulin, lysophosphatidic acid, and glucagon increased differentiation of bovine SCs grown in serum-free conditions and that acetylcholine enhanced maturation and fusion. Chal et al. (2016) developed a protocol for in vitro generation of myofibers and SCs from human PSCs that relied on the sequential application of optimized culture media formulations. Based on the zebrafish blastomere screen described above, Xu et al. (2013) designed a differentiation cocktail containing FGF2, the adenylate cyclase activator forskolin, and the GSK3beta inhibitor BIO, which differentiated human iPSCs into multinucleated myotubes. Tanaka et al. (2013) demonstrated the direct conversion of human iPSCs to myocytes by expression of *MYOD1*, and Watanabe et al. (2011) noted that mouse myoblast-derived iPSCs may have improved myogenic differentiation potential compared to those derived from other cell types. Several studies have reported myogenic differentiation of MSCs in response to coculture with, or treatment with conditioned media from, myogenic cell lines (Stern-Straeter et al. 2014; Patruno et al. 2017; Korovina 2019). Because the overall process of myogenesis shares many molecular details between species, efforts to produce better methods for differentiating fish muscle should also explore the hypothesis that these same protocols might be successful in fish.

### Signals Regulating the Decision to Become Fast or Slow Fibers

**Fig. 4:**
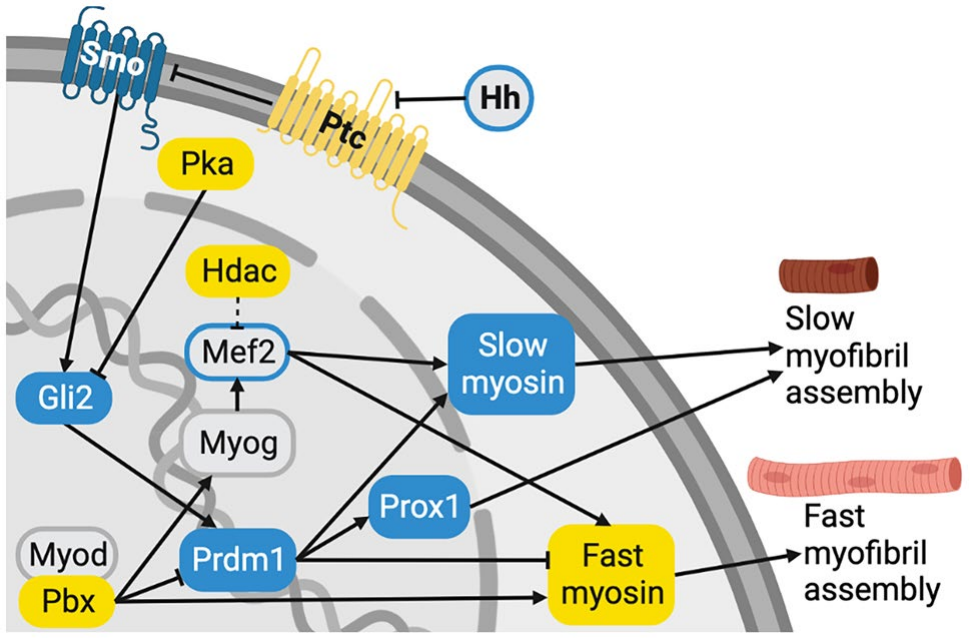
Proteins involved in specifying slow versus fast muscle fate in fish (Du et al. 1997; Barresi et al. 2000; Du and Dienhart 2001; Roy et al. 2001; Hinits and Hughes 2007; Potthoff et al. 2007; Potthoff and Olson 2007; Yao et al. 2013). Proteins shown in blue primarily promote development of slow muscle, and those shown in yellow primarily promote a fast muscle fate. Those that play an important role in both fiber types are shown in gray, with blue outlines indicating those playing a substantially greater role in promoting a slow muscle fate. Dashed lines indicate mechanisms that have been demonstrated in other model systems but not in fish

Fish white muscle, made up of fast fibers, is generally perceived most positively in a food context (Listrat et al. 2016). However, red muscle in the correct ratio and geometry may be required for specific products such as hamachi or to produce desirable flavor compounds during cooking. Because several transcription factors have dual roles in promoting cell differentiation and maturation and influencing muscle fiber type specification (Hinits and Hughes 2007; Potthoff and Olson 2007), monitoring fast and slow muscle marker expression during bioprocesses development will be necessary to avoid unwanted organoleptic effects. Proteins thought to be involved in fiber type specification are outlined in Fig. 4.

Fast and slow muscle progenitors can be distinguished within the zebrafish presomitic mesoderm (Devoto et al. 1996; Coutelle et al. 2001; Jackson and Ingham 2013). However, in some instances, cells’ commitment toward a specific fiber type could be reprogrammed with relative ease (Potthoff et al. 2007). Slow fibers develop from the notochord-adjacent adaxial cells, while the paraxial cells become fast muscle (Hatta et al. 1991; Devoto et al. 1996). Adult fish slow fibers are mononucleated, most of which are found in a thin layer below the skin as the “superficial slow fibers,” while those that form the horizontal myoseptum are derived from precursors known as the “muscle pioneers” (Hatta et al. 1991; Roy et al. 2001). The spatial separation of slow and fast muscle means that slow- and fast-fated myoblast cultures can be initiated simply by careful dissection of the muscle (Duran et al. 2020).

Many studies have implicated the Hh pathway, especially notochord-derived Shh, in steering zebrafish muscle cells toward maturation into slow fibers (Weinberg et al. 1996; Barresi et al. 2000; Du and Dienhart 2001; Coutelle et al. 2001; Osborn et al. 2011). Slow muscle development in vivo can be prevented by blocking Shh signaling (Blagden et al. 1997; Du et al. 1997). Together with the fact that zebrafish adaxial cells express fast muscle-specific genes prior to somite formation (Xu et al. 2000; Hinits and Hughes 2007), this may imply that myocytes become fast fibers by default unless exposed to sufficient Shh levels. Cell differentiation into slow fibers failed when gilthead seabream embryos were treated with forskolin, an adenylate cyclase activator, presumably due to Hh pathway inhibition (Tan and Du 2002). However, the effects of Hh signaling depend on dosage and timing, and in some cases, Hh may also promote differentiation into fast muscle fibers (Wolff et al. 2003; Feng et al. 2006).

Downstream signals mediating the effects of Hh signaling on zebrafish slow muscle include smoothened (Smo) (Barresi et al. 2000), glioma-associated oncogene (Gli2) (Du and Dienhart 2001), cyclin-dependent kinase inhibitor 1C (Cdkn1c) (Osborn et al. 2011), Prdm1a, and Prox1 (Roy et al. 2001). Prdm1a is antagonized by the transcription factor Pre-B-cell leukemia transcription factor (Pbx) (Yao et al. 2013). Pbx2 and Pbx4 cooperate with MyoD to promote differentiation into fast fibers (Maves et al. 2007).

Class II HDACs promote cells’ differentiation into fast muscle fibers in mice by repressing the transcription factor myocyte enhancer factor 2 (MEF2) (Potthoff et al. 2007). It is unknown whether this phenomenon exists in fish and, if so, which Mef2 and Hdac isoforms are involved. MEF2 regulates the differentiation of multiple cell types (Potthoff and Olson 2007) and zebrafish Mef2 plays a role in both fast and slow fiber maturation (Hinits and Hughes 2007). Therefore, it might be possible to control fiber type specification by manipulating this pathway in committed cell types such as SCs, though the effects may be sensitive to expression levels, isoforms (Ticho et al. 1996), splice variants, transcriptional states, or other factors.

## Adipogenesis in Fish

Skeletal muscle-associated adipocytes are present in teleost species from multiple orders. In rainbow trout, red seabream, and pacific herring, adipocytes can be found in white muscle, myosepta, slow muscle, and in gaps between muscle bundles (Kaneko et al. 2016). In pelagic fish such as salmon, Pacific herring, and Pacific saury, intracellular lipid deposition can also be found in red muscle (Kaneko et al. 2016).

Adipocytes can differentiate from cells including MSCs (Ytteborg et al. 2015; Salmerón et al. 2016; Bou et al. 2017), FAPs (Reiss et al. 2021), preadipocytes, and—at least in mice—SCs (Asakura et al. 2001), as well as possibly from fibroblast-like cells (Tsuruwaka and Shimada 2022). The FAP differentiation process is conserved among species and potentially can be applicable for fish species. However, during fish adipogenesis, the timeline of events should be adjusted when translating such knowledge (Li et al. 2020).

### Markers of Adipogenic Differentiation

**Fig. 5:**
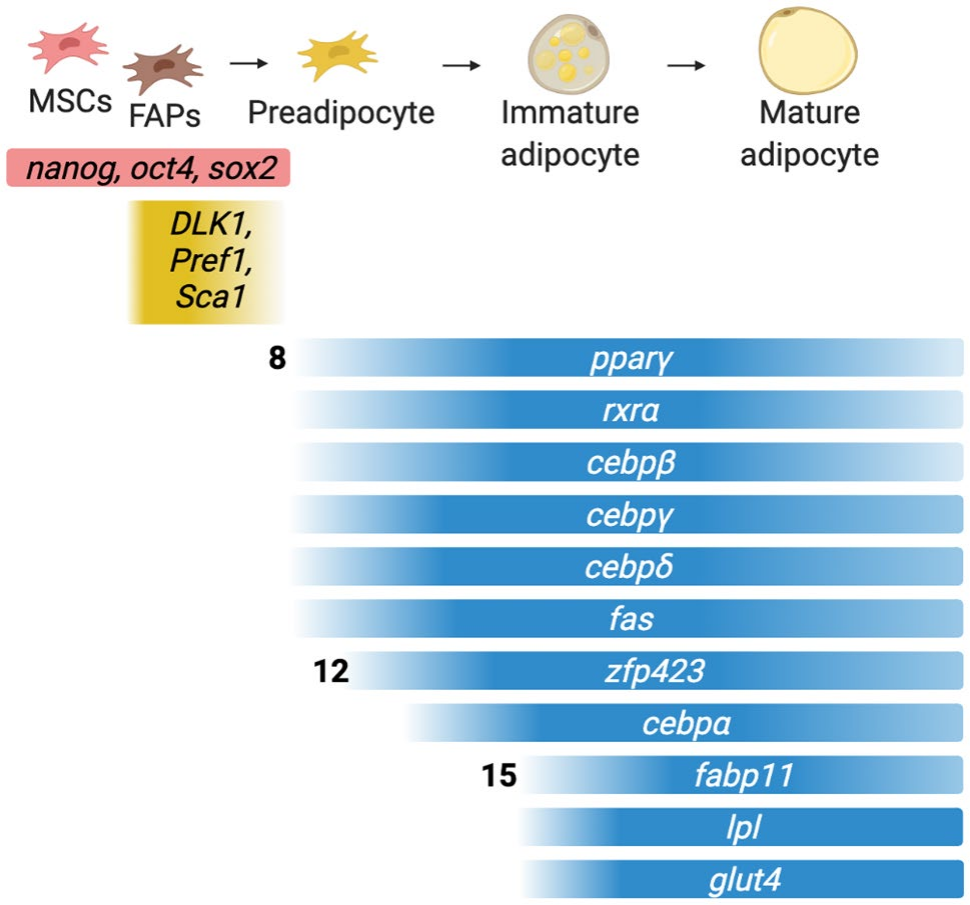
Genes involved in fat differentiation and maturation in fish (Vegusdal et al. 2003; Oku and Umino 2008; Hesslein et al. 2009; Todorcević et al. 2010; Huang et al. 2012a; Mota de Sá et al. 2017; Liu et al. 2018; Salmerón 2018). Genes are ordered according to their approximate order of activation during adipogenic differentiation. Numbers to the left of some rows indicate the timing of activation within the zebrafish somite in hours post-fertilization (Den Broeder et al. 2015). Genes fading out to the right are downregulated in mature fat tissue relative to precursors. Red indicates genes associated with zebrafish MSCs (Fathi et al. 2019) and gold indicates those primarily associated with FAP appearance in mammalian systems (Li et al. 2020). Blue indicates the genes related to adipogenesis from the preadipocyte state until achieving a mature adipocyte

Terminal differentiation of adipocytes can be observed as an increase in lipid accumulation, often visualized using stains such as oil red O (Vegusdal et al. 2003)—a standard readout of adipogenic differentiation—and characterized by increased expression of genes related to lipid metabolism (e.g., *pparg*, *cebpb*, and *fas*; Fig. 5).

Salmon cells undergoing adipogenesis begin to express *c/ebpβ*, *pparγ*, *fas*, *c/ebpα*, *bone morphogenetic protein 4* (*bmp4*), *lipoprotein lipase* (*lpl*), and *c/ebpδ* in approximately that order (Todorcević et al. 2010; Huang et al. 2010). Pparγ, C/ebP/α, and leptin expression have been previously reported in differentiated salmon adipocytes (Vegusdal et al. 2003), as have *pparγ*, *c/ebpα*, *c/ebpγ*, and *fas* in differentiating preadipocytes from grass carp (Liu et al. 2018). The most useful markers to distinguish preadipocytes from cells that have already begun adipogenesis are those whose expression can be allocated to specific stages. These include *c/ebpγ*, *fas* (Liu et al. 2018), *c/ebpα* (Todorcević et al. 2010; Huang et al. 2010), *fatp1* (Huang et al. 2010), and *bmp4* (Todorcević et al. 2010), which become expressed during intermediate stages of differentiation, and *fabp11* (Huang et al. 2010) during later stages.

### Signals Initiating Adipogenic Differentiation

**Fig. 6:**
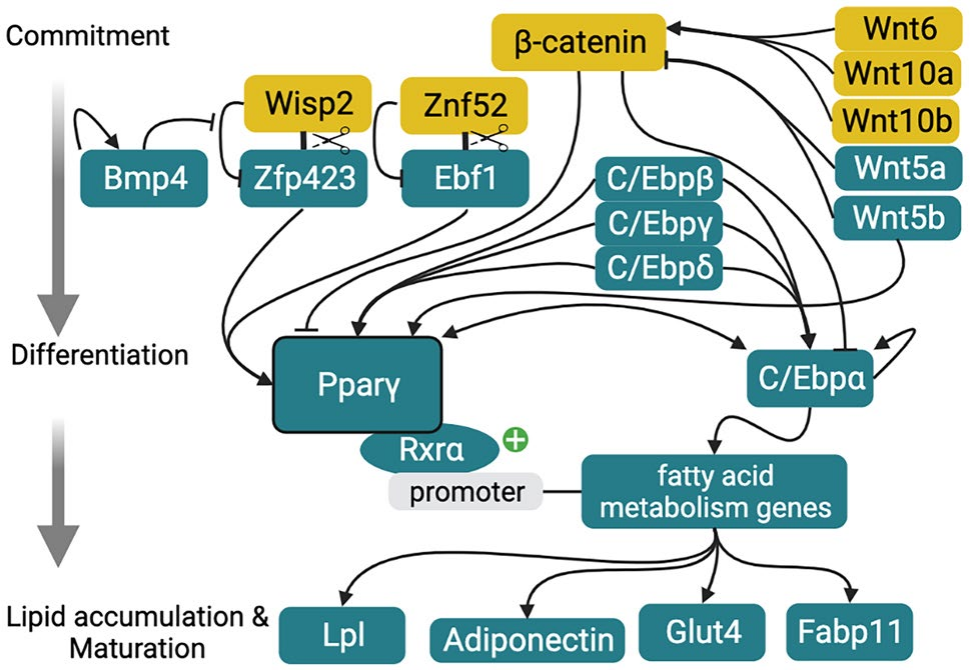
Signaling pathways involved in adipogenic commitment, differentiation, and maturation in fish (Ross et al. 2000; Vegusdal et al. 2003; Oku and Umino 2008; Christodoulides et al. 2009; Hesslein et al. 2009; Todorcević et al. 2010; Cawthorn et al. 2012; Huang et al. 2012a; Ytteborg et al. 2015; Mota de Sá et al. 2017; Liu et al. 2018; Salmerón 2018). Gold indicates signals primarily associated with maintenance of quiescent or proliferative states and teal indicates those primarily associated with differentiation

As with muscle, various genes and signaling pathways are involved in activating or repressing adipogenesis in fish (Fig. 6).

#### Peroxisome Proliferator-Activated Receptors (Ppar ɑ, β, and γ)

Three Ppar isoforms (ɑ, β, and γ) are present in teleost fish, and these nuclear receptors have important roles in adipogenesis (Cruz-Garcia et al. 2009). Pparγ is the major adipogenic regulator that leads to transcriptional activation of genes that facilitate lipid storage and fatty acid metabolism (Salmerón 2018). This occurs when Pparγ attaches to the cis-retinoic acid receptor alpha (Rxrα) and binds the promoters of those genes. Pparγ is required for the in vitro differentiation of Atlantic salmon adipocytes and cooperates with other regulators such as C/Ebpα (Vegusdal et al. 2003). Oku and Umino (2008) supported that Pparγ is essential in adipocyte differentiation, although in red sea bream cultures this marker was not directly linked to adipocyte differentiation. While in humans (and mice) PPARγ was reported to have two isoforms (PPARγ1 and PPARγ2), in zebrafish there is only one (Pparγ1) (Den Broeder et al. 2015; Wafer et al. 2017).

#### CCAAT/Enhancer-Binding Proteins (C/Ebpα, C/Ebpβ, C/Ebpγ, and C/Ebpδ)

C/Ebps are a family of six transcription factors with several domains that allow DNA recognition followed by gene regulation, where C/Ebpα, β, γ, and δ specifically regulate genes that promote adipogenesis. The expression of each transcription factor is dependent on the species, but usually in mammals, C/Ebpβ and C/EbpPδ are induced in the early stages of adipogenesis and together induce expression of C/Ebpα (Mota de Sá et al. 2017). In fish, C/Ebpα and C/Ebpβ appear to have important roles in zebrafish embryonic development (Lyons et al. 2001), and both have significant protein homology to both human and mouse orthologs (Imrie and Sadler 2010). However, in studies performed with salmon preadipocytes, *c/ebpα* is expressed relatively late during adipogenesis (Todorcević et al. 2010; Huang et al. 2010). Moreover, C/Ebpα can impact its own production and the expression of Pparγ through a positive feedback mechanism (Rosen et al. 2002; Mota de Sá et al. 2017). In studies using carp preadipocytes, a high expression of *c/ebpγ* was also detected (Liu et al. 2018).

#### Zinc-Finger Protein 423 (Zfp423)

Zfp423 is a transcription regulator required in early adipogenic commitment with limited sequence variation among vertebrates (Gupta et al. 2010; Hamilton 2020). Zfp423 becomes active when two inhibitory complexes, Wnt1 inducible signaling path-way protein 2 (WISP2)–Zfp423 and ZNF52–Ebf1, disassociate, allowing Zfp423 and Ebf1 to enter the nucleus and activate Pparγ transcription (Hesslein et al. 2009; Hammarstedt et al. 2013). This process can be blocked by bta-miR-23a via direct targeting of Zfp423 (Guan et al. 2017). Therefore, Zfp423 expression is essential to initialize determination and to allow the commitment of preadipocytes. In fact, Zfp423 knockdown in mouse embryos resulted in impaired adipose tissue development (Gupta et al. 2010; Shao et al. 2017). The same knockdown in bovine adipose cells prevented their differentiation in vitro, while overexpression improved their adipogenic potential (Huang et al. 2012b). Moreover, studies using immortalized adipogenic progenitor cells from bovine muscle SVF found that Zfp423 regulation is required for intramuscular fat development and that highly adipogenic cells (cells that stain intensely with Oil-Red-O) expressed Zfp423, Pparγ, C/Ebpα, and C/Ebpβ (Huang et al. 2012b).

#### FGF-1 and FGF-2

Supplementation with FGF-1 and FGF-2 can positively impact adipogenic differentiation. In human AMSCs, FGF-1 supplementation before adipogenic induction with a hormone cocktail increased mRNA expression of *C/EBPα* in a dose-dependent manner (Kim et al. 2015). Other studies also reported that FGF-2 increases proliferation and directly affects adipogenic differentiation by inducing the expression of PPARγ (Kakudo et al. 2007). The mRNA expression of CEBPα was inversely correlated with FGF-2 concentration (Kim et al. 2015). Besides media supplementation, incorporation of FGF-2 in microspheres composed of gelatin enabled preadipocytes to differentiate into adipose tissue in a mouse model (Tabata et al. 2000; Yun et al. 2010).

#### TGF-β and BMP4

The TGF-β pathway, the conservation of which was investigated by Huminiecki et al. (2009), is composed of TGF-β and BMP signaling ligands, surface receptors, and SMAD signaling proteins. TGF-β ligands attach to TGF-β receptors that become phosphorylated, consequently leading to phosphorylation of SMAD3. Phosphorylated SMAD3 can cross the nuclear membrane and inhibit the expression of CEBPs and PPARγ (Li and Wu 2020). Consequently, early TGF-β signaling appears to promote adipogenic commitment but also to inhibit adipogenic differentiation. TGF-β is somewhat conserved across species, with peptide sequences from rainbow trout strongly aligned with those of striped bass (Harms et al. 2000). Carp Tgf-β_2_ appears to be loosely related to avian and mammalian TGF-β_2_ isoforms.

BMP4, a member of the TGF-β superfamily, is secreted by differentiated preadipocytes and induces the adipogenic commitment of precursor cells (Gustafson et al. 2015). When BMP4 is released, it activates a receptor that promotes the dissociation of the inhibitory WISP2–ZFP423 complex (Hammarstedt et al. 2013), thereby activating PPARγ transcription. In addition, cultures of Atlantic salmon primary adipose SVF cells demonstrated an increased expression of Bmp4 after chemical induction of adipocyte differentiation (Todorcević et al. 2010).

#### Wnt Pathways Regulate Adipogenesis

The Wnt family of signaling proteins has an important role in regulating tissue maintenance and remodeling. Activation of the Wnt pathway and signaling through β-catenin represses adipogenesis by inhibiting the expression of PPARγ and CEBPα (Christodoulides et al. 2009). One mechanism by which Wnt family proteins prevent activation of PPARγ is thought to be activation of WISP2, the aforementioned inhibitor of Zfp423 (Hammarstedt et al. 2013). As mentioned in the “Preadipocytes” section, the Wnt/β-catenin pathway has maintained preadipocytes in an undifferentiated and proliferative state in carp (Liu et al. 2018). In cultures of grass carp preadipocytes, this was observed after 7 days of DHA (100 µM) supplementation, where carp adipocytes had decreased expression of C/Ebpα, Pparγ, C/Ebpγ, and Fas (Liu et al. 2018). In 3T3-L1 preadipocytes, a similar treatment resulted in expression of Wnt1, thus inhibiting the activation of *PPARγ* gene expression (Moldes et al. 2003).

In addition to this canonical pathway, Wnt family members can also activate non-canonical signaling through Wnt5a and Wnt5b, both of whose gene expression in salmon MSCs increases throughout the adipogenic process (Ytteborg et al. 2015). In 3T3-L1 preadipocytes, Wnt5b promotes the adipogenic process by down-regulating β-catenin (Kanazawa et al. 2005). Both Wnt5a and Wnt5b have been suggested to promote early adipogenesis by enhancing the expression of PPARγ (Kanazawa et al. 2005; van Tienen et al. 2009). Besides those factors, Wnt6, Wnt10a, and Wnt10b are also early regulators of adipogenic commitment and their overexpression leads to a decrease in the mRNA expression of *PPARγ* and *C/EBPα*, mediated by β-catenin (Cawthorn et al. 2012). Moreover, Wnt10b helps maintain the preadipocyte state, and its blockage induces transdifferentiation of myoblasts into adipocytes (Ross et al. 2000). While most of these studies are on mammalian cell models, their conservation among different animals may inspire strategies to induce adipogenesis in fishes.

#### IGF-1 and Insulin

Insulin and insulin-like growth factor 1 (IGF-1) are important adipogenic regulators, as demonstrated in vivo by reductions in adipose tissue formation in transgenic mice lacking insulin and/or IGF-1 receptors (Boucher et al. 2016), and also influence preadipocyte proliferation (see Sect. 2.5). While both insulin and IGF-1 increased differentiation and lipid accumulation of gilthead seabream preadipocytes, a stronger effect was observed from IGF-1 (Salmerón et al. 2013).

#### Hedgehog (Hh) Pathway

The Hh pathway regulates cell fate determination, proliferation, migration, polarity, and gene expression. In adipogenesis, the Hh pathway is involved with expression of PPARγ, leading to possible alterations in the fates of precursor cells. In 3T3-L1, NIH-3T3 cells, and porcine AMSCs, Hh pathway activation inhibits adipogenic differentiation (Fan et al. 2018). Such inhibition was also observed during fat formation in 3T3-L1 preadipocytes and in the *Drosophila* fat body, suggesting a conserved role for the Hh pathway as an adipogenic regulator in vertebrates and invertebrates (Suh et al. 2006). Even with less information on the impact of Hh pathway modulation on fish fat tissue, Wynne et al. (2021) considered that this pathway is also associated with cell fate and proliferation in teleost fishes.

#### MicroRNAs

MicroRNAs (miRNAs) are short RNAs that regulate gene expression via multiple mechanisms and have a well-characterized role in regulating mammalian adipogenesis (Romao et al. 2011), including a potential role in bovine intramuscular and subcutaneous fat (Guo et al. 2017; Mir et al. 2020). As of March 3, 2022, miRBase—a database of published miRNA sequences (Griffiths-Jones 2004; Griffiths-Jones et al. 2006, 2008; Kozomara and Griffiths-Jones 2011, 2014; Kozomara et al. 2019)—contained sequences of 1917 precursors and 2654 mature mRNAs from human, 1064 precursors/1025 mature from bovine, 371 precursors/497 mature from salmon, and 355 precursors/373 mature from zebrafish.

Various miRNAs have been identified that promote or inhibit adipogenesis, including in fish. MiR-143 has been characterized as a marker of lipid deposition in rainbow trout, and some evidence suggests a mechanistic role in promoting adipogenesis via inhibition of the α/β hydrolase-domain 5 (*abhd5*) gene (Mennigen et al. 2013). Similarly, miR-150-4p expression in chickens promoted differentiation of intramuscular adipocytes by targeting retinoid X receptor gamma (Zhang et al. 2018).

Depleting miR-27b in zebrafish increased adipocyte hyperplasia, lipid accumulation, and expression of adipocyte-related genes including Pparγ and C/Ebpɑ, indicating that it serves as a negative regulator of adipogenesis (Hsu et al. 2018). This is consistent with findings in other vertebrates; for example, in human MSC-derived adipocytes, miR-27b has been shown to directly suppress PPARγ and to inhibit lipid accumulation and adipogenesis-associated marker gene expression when overexpressed (Karbiener et al. 2009). In both mouse and human cells, miR-182 inhibits adipogenesis by targeting C/EBPɑ and is downregulated temporarily during early adipogenesis (Dong et al. 2020).

### Terminal Differentiation and Accumulation of Lipid Droplets (i.e., Lipogenesis)

A lipid source (e.g., oleic acid or a lipid cocktail) is routinely added to the medium for adipogenic differentiation of fish preadipocytes (Vegusdal et al. 2003; Todorcević et al. 2010; Liu et al. 2018; Salmerón 2018), though it has also been reported that long-chain omega-3 fatty acids may inhibit differentiation (Huang et al. 2010; Liu et al. 2018). Differentiation of Atlantic salmon preadipocytes was induced by a cocktail of medium supplements including insulin, dexamethasone, biotin, triiodothyronine, pantothenate, isobutylmethylxanthine (IBMX), fatty acids, and cholesterol (Todorcević et al. 2010) (Table 3).

**Table 3:** Examples of protocols shown to successfully induce or accelerate adipogenic commitment/differentiation in fish cultures

Species and cell type	Treatment	Effect	Citation
Red sea bream primary culture of preadipocytes from the SVF	DMEM/Ham’s F12 with 65 mM NaCl and 10% FBS (proliferation medium) Proliferation medium plus ITS mixture (i.e. bovine insulin, transferrin, sodium selenite), and hydrocortisone (differentiation medium)	Lipid droplets in the cytoplasm were observed after 10 days with differentiation medium, associated with high expression of *lpl* and *fas*. Results showed that Pparγ is required for adipocyte differentiation	(Oku and Umino 2008)
Gilthead sea bream primary culture of preadipocytes from the SVF	DMEM with 60 mM NaCl, 1% A/A and 10% FBS (proliferation medium) Proliferation medium plus insulin, IBMX, dexamethasone, and a lipid mixture (cholesterol and fatty acids from cod liver oil) (differentiation medium)	Cells reached confluence using proliferation medium at day 8 Differentiation medium induced a rounded cell shape with an enlarged cytoplasm that became filled with lipid droplets IGF-I is more efficient than insulin enhancing differentiation	(Salmerón et al. 2013)
Atlantic salmon primary culture of preadipocytes from the SVF	L15, 10% FBS, L-glutamine, 10 mM HEPES, and antibiotics (proliferation medium) Proliferation medium plus dexamethasone, biotin, triiodothyronine, pantothenate, isobutylmethylxanthine, insulin, and a lipid mixture of cholesterol and cod liver oil fatty acids (differentiation medium)	Cells reached confluence using proliferation medium at day 7 2 days after addition of the differentiation medium, cells changed to a more rounded shape *pparγ* was up-regulated on day 4. *c/ebp​​β*, was up-regulated until confluence and *c/ebpδ* and *c/ebpα* increased after the addition of differentiation medium	(Todorcević et al. 2010)
Atlantic salmon primary culture of preadipocytes from the visceral adipose tissue	DMEM, 10% FBS, 2 mM L-glutamine, 10 mM HEPES (proliferation medium) Proliferation medium plus a lipid mixture (cholesterol, cod liver oil fatty acids, polyoxyethylene sorbitan monooleate, and D-α-tocopherol acetate (differentiation medium)	Lipid mixture promoted a rapid and extensive differentiation. Pparγ was expressed in the nuclei early in the differentiation. Pparγ cooperates with C/Ebpα	(Vegusdal et al. 2003)
Rainbow trout primary culture of preadipocytes	L-15, 10% fetal bovine serum, 2 mM L-glutamine and 1% A/A solution (proliferation medium) Proliferation medium plus insulin, IBMX, dexamethasone, and a lipid mixture (differentiation medium)	High expression of Pparγ in differentiating adipocytes at day 15 Genes involved in energy production, lipid and carbohydrate metabolism, lipid droplet formation and Rxr were detected in terminal differentiation	(Bou et al. 2017)

In differentiated adipocytes from Atlantic salmon, the expression of genes for adipokines adipsin and visfatin coincides with the accumulation of lipid droplets (Todorcević et al. 2010). An increase in expression of NADPH-related genes, such as *glucose-6-phosphate dehydrogenase* (*g6pd*) or *6-phosphogluconate dehydrogenase* (*pgd*), has been reported in terminal differentiation of salmon adipocytes, which is aligned with the need of NADPH for triacylglycerol/lipid production and accumulation (Todorcević et al. 2010).

Insulin decreased lipolysis in the mature adipocytes, whereas Tnfɑ increased this process (Wang et al. 2012b). However, while another study in adipocytes isolated from gilthead seabream found that insulin decreased lipolysis in some experiments, these effects were inconsistent (Albalat et al. 2005).

Tnf-related genes are down-regulated in salmon SVF cells upon adipogenic induction, though up-regulated at initial stages before reaching confluence (Todorcević et al. 2010). Further research in gilthead seabream mesenteric adipocytes suggests that Tnfɑ regulation of adipogenic factors varies amongst fat and lean phenotypes (Cruz-Garcia et al. 2009). In this study, the authors reported that Tnfɑ had lipolytic effects and reduced lipid accumulation characterized by Pparγ downregulation in adipocytes from lean fish. In contrast, the adipocytes from fat specimens had Pparβ-mediated lipolytic effects or no apparent changes from Tnfɑ supplementation.

## Fish Connective Tissue, Vascular Tissue, and Skin

While muscle and fat cells are the main contributors to the organoleptic and nutritional properties of meat, connective tissue also plays an essential role in both the mechanical properties of the tissue and the changes to those properties during the cooking process (Listrat et al. 2016). In the context of CS, the scaffold might fully or partially substitute this role. Fibroblasts or other extracellular matrix-secreting cell types could also be incorporated. Fortunately, fibroblast-like cells and cells derived from fin tissue are abundant among fish cell lines (Thangaraj et al. 2021) and are easily isolated and cultured. Future research into the use of fibroblasts in CS should focus on identifying optimal culture conditions for serum-free co-cultures of fibroblasts with myogenic and adipogenic cells and investigating the effects of these cell types on one another’s proliferation and differentiation.

Bricard et al. (2014) identified a population of extracellular matrix-secreting cells in the myosepta of trout embryos that appeared analogous to mammalian tenocytes and expressed the tenocyte marker scleraxis. This population was later shown to be dependent on Hh signaling, and its loss was shown to lead to a muscle detachment phenotype (Ma et al. 2018). This latter observation suggests that tenocytes might substantially contribute to fish’s mechanical and organoleptic properties.

Although vascularization of tissue is not likely to contribute meaningfully to taste or texture (Listrat et al. 2016), some scaffolding strategies might require the presence of endothelial or smooth muscle cells. Adding pro-angiogenic factors (Huang et al. 2012a) at strategic points during the bioprocess might facilitate the creation of vascularized tissues from a single multi- or pluripotent cell line.

For many CS product applications, a lack of skin will be an advantage (Rubio et al. 2019a). In cases where skin is desirable, co-cultures of fibroblasts and scale-derived epithelial cells may be used (Rakers et al. 2011), likely in conjunction with methods to encourage slow muscle growth (see Section 3.4).

## Myogenesis and Adipogenesis in Aquatic Invertebrates

Myogenesis and adipogenesis are even less well understood in aquatic invertebrates than in fish. Table 4 summarizes some of the molecular players and pathways thought to be involved in invertebrate myogenesis.

**Table 4:** Genes, proteins, and molecular pathways implicated in invertebrate myogenesis

Gene, protein or pathway	Effect/Observation	Animal & cell type or system	Reference
*Twist*/Twist (**arthropods**)	Initial mesoderm patterning	**Insects** Fruit fly embryos	(Bothe and Baylies 2016)
		**Crustaceans** Isopod crustacean embryos	(Price and Patel 2008)
		Penaeid shrimp embryos	(Wei et al. 2016)
*Twist2/*Twist2 (**bivalves**)	Implicated in early myogenic differentiation of MSCs	Scallop adductor muscles	(Sun et al. 2021)
*Nautilus*/Nautilus (**insects**)	Founder cell positioning Initiating somatic myogenesis	Fruit fly embryos	(Zhang et al. 1999)
		Fruit fly fibroblasts	(Wei et al. 2007)
		Fruit fly cardioblasts	(Keller et al. 1997)
*Mef2/*Mef2 (**arthropods**)	Terminal differentiation and fusion	**Insects** Fruit fly embryos	(Bour et al. 1995; Taylor ﻿^2006^; Bryantsev et al. 2012),
		**Crustaceans** Isopod crustacean embryos	(Price and Patel 2008)
		Penaeid shrimp embryos	(Wei et al. 2016)
*Pax3/*Pax3 (**crustaceans**)	Implicated in embryonic and regenerative myogenesis	Crayfish embryos, abdominal muscle and regenerating limbs	(White et al. 2005)
	Active in satellite-like cells during myogenesis	Isopod crustacean embryos and regenerating limbs	(Konstantinides and Averof 2014)
*Mstn/*Myostatin (**multiple lineages**)	**Crustaceans** Promotes muscle atrophy	Lobster claw muscle	(MacLea et al. 2010)
	Potentially promotes myogenesis	Shrimp abdominal muscle Crab thoracic muscle	(De Santis et al. 2011) (MacLea et al. 2012)
	Potentially inhibits myogenesis	Shrimp muscle	(Yan et al. 2020; Wang et al. 2021)
	**Gastropods** Upregulation of insulin pathway	Abalone muscle tissue	(Carrera-Naipil et al. 2016)
Ecdysone (**arthropods**)	**Insects** Activates Twist and Mef2	Fruit fly embryos	(Lovato et al. 2005)
	Initiates terminal differentiation	Grasshopper myoblasts	(Baryshyan et al. 2012)
	**Crustaceans** Stimulates muscle protein synthesis via:		
	- Suppression of myostatin	Land crab claw muscle	(Covi et al. 2010)
	- Activation of mTOR via Rheb	Land crab claw muscle	(MacLea et al. 2012)
*NK4*/NK4 (**cephalopods**)	Myogenic progenitor determination	Cuttlefish embryos	(Navet et al. 2008)
*Mox*/Mox (**gastropods**)	Muscle lineage determination of mesoderm	Abalone embryos	(Hinman and Degnan 2002)
Hedgehog pathway (**mollusks**)	**Cephalopods** Myogenic progenitor proliferation	Cuttlefish myoblasts	(Grimaldi et al. 2008)
	**Bivalves** Highly expressed during myogenesis stage	Oyster embryos	(Li et al. 2018)
mTOR/insulin/P13K-Akt pathways (**bivalves**)	Increased muscle growth	Clam whole tissues	(Nie et al. 2021)
		Oyster whole tissues	(Choi et al. 2018; Kim and Choi 2019; Li et al. 2021)

### Crustaceans

#### Myogenesis

In many crustaceans, muscle fiber types appear to exist on a spectrum rather than in distinct fast and slow categories, with each type having a unique expression profile of myofibrillar proteins and isoforms (Medler and Mykles 2003). Relative composition of fast or slow types, and that of tissues undergoing protein synthesis or degradation, vary continually to accommodate the remodeling required for ongoing molt cycles (Mykles 1997). These variations may have implications for cultivated crustacean meat.

Early myogenesis appears to be similar to that of the fruit fly *Drosophila melanogaster* where “founder” cells migrate from the mesoderm and fuse with undifferentiated myoblasts to form muscle progenitors (Kreissl et al. 2008; Jirikowski et al. 2010; Harzsch and Kreissl 2010). Myogenesis also occurs during appendage regeneration in both groups, though in crustaceans, this process more closely replicates embryogenesis where a developing blastema forms the pool of undifferentiated cells from which myogenic precursors emerge (Hopkins et al. 1999; Hopkins and Das 2015).

Little is known about the molecular drivers of myogenesis in crustaceans; however, it is recognized that there are likely similarities with *Drosophila* (Mykles and Medler 2015), where the key myogenic transcription factors are Twist, Nautilus, and Mef2 (Taylor 2006). Twist, most similar in function to vertebrate MyoD, is expressed early and is important for initial determination and patterning of the mesoderm, and then for formation of early myogenic progenitors and founder cells (Taylor 2006; Bothe and Baylies 2016). Nautilus, more similar to MyoD in sequence (Taylor 2006), is expressed later and is important for founder cell patterning (Wei et al. 2007). Nautilus has also been shown to initiate the myogenic program in adult fibroblasts (Zhang et al. 1999) and cardioblasts using the GAL4-targeted system (Keller et al. 1997). Mef2 works synergistically with Twist and Nautilus, as it does with the vertebrate MRFs, and is important for cells’ terminal differentiation and fusion (Bour et al. 1995; Taylor 2006; Bryantsev et al. 2012).

In the isopod crustacean *Parhyale hawaiensis*, expression of Twist is also evident at the mesodermal patterning stage and induces Mef2, which is required for later muscle determination and differentiation (Price and Patel 2008). In various penaeid shrimp, Mef2 appears to be expressed earlier than Twist but is also necessary for myogenic determination and differentiation (Wei et al. 2016). Studies on crustacean Nautilus were not found; however, in the crayfish *Cherax destructor*, Pax3 is implicated in embryonic myogenesis as well as after molting and during appendage regeneration (White et al. 2005). A regenerative role for Pax3 has also been seen in *Parhyale* (Konstantinides and Averof 2014).

Myostatin in crustaceans appears to have multiple functions beyond myogenic regulation and its direct effect on myogenesis remains unclear (Mykles and Medler 2015; Yan et al. 2020). Studies on shrimp show both a positive effect (De Santis et al. 2011) and a negative effect (Yan et al. 2020; Wang et al. 2021), and its effect on different muscle types (thoracic versus claw) within the same crab species also appear to be contradictory (MacLea et al. 2012).

#### Molt Hormones and Growth Factors

**Fig. 7:**
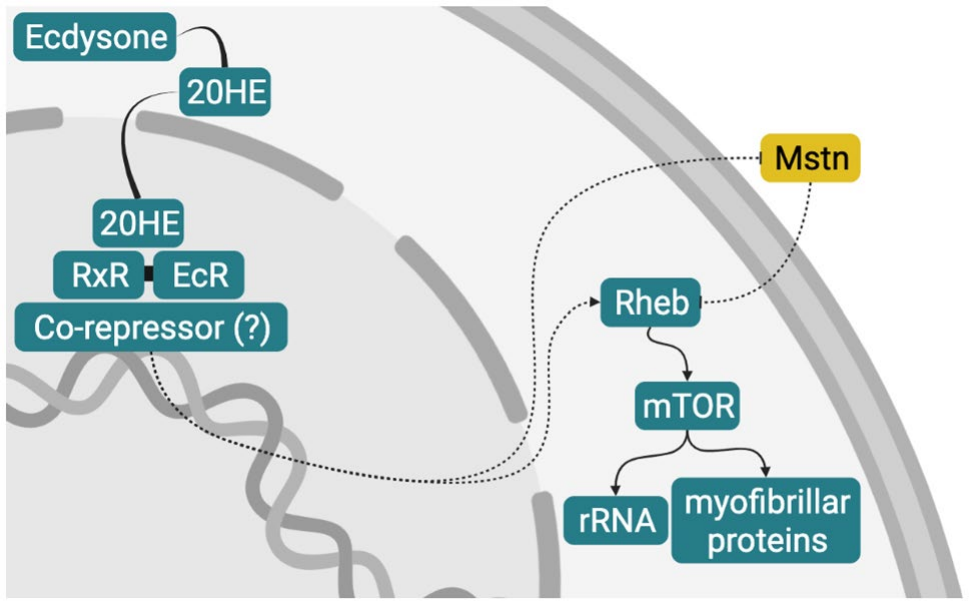
Potential signaling pathway of the molt hormone ecdysone in land crab claw muscle during the premolt stage (Covi et al. 2010; MacLea et al. 2012). The active form, 20HE, binds to the EcR-RxR nuclear receptor and activates Rheb, either directly or through the repression of myostatin (possibly via a corepressor). Rheb is a major activator of mTOR, known to stimulate protein translation. Gold indicates where inactivation is required for myogenic protein synthesis, and teal indicates activation. Dashed lines indicate steps where the exact mechanism(s) involved are unclear

Molting is intrinsically linked to muscle growth and development in all arthropods, driven by ecdysteroids such as ecdysone (Mykles and Medler 2015). In *Drosophila*, ecdysone appears to induce myogenesis through activation of Mef2 via Twist (Lovato et al. 2005). Several in vitro insect studies have shown ecdysone media supplementation induces terminal differentiation in various myogenic cells, particularly myoblasts (Baryshyan et al. 2012; Rubio et al. 2020). In crustaceans, high titers of ecdysteroids have been shown to stimulate protein synthesis in different muscle types across the molt cycle, although apparently through varying pathways such as myostatin or Rheb (Mykles 1997; Covi et al. 2010; MacLea et al. 2012). Figure 7 outlines these potential pathways in the claw muscle of premolt land crab. This hormone is readily accessible and thus an ideal candidate with which to begin crustacean muscle differentiation experiments.

The effect of growth factors has also been observed on crustacean muscle growth, with IGF supplementation both in vitro and in vivo showing increases in muscle protein synthesis in crayfish (Chaulet et al. 2012; Jayesh et al. 2015). Other studies have shown varying results using different recombinant growth factors, with one review highlighting that growth factors obtained from more species-relevant serums or tissue extracts are likely to have more promising results (Ma et al. 2017). Once identified, crustacean growth factor homologs could potentially be overexpressed to induce myogenesis or made recombinantly and used in media supplementation.

#### Crustacean Fat Synthesis

Rather than intramuscular adipocytes as in vertebrates, crustacean muscle lipid content appears to be derived mainly from cell membrane phospholipids (PL) and sterols, with a large component of the PLs being long chain (lc) polyunsaturated fatty acids (PUFA), such as the nutritionally important omega-3 fats, eicosapentaenoic acid (EPA) and DHA (Chapelle 1977; Zhao et al. 2015; Shu-Chien et al. 2017; Lu et al. 2020). Although some lc-PUFA synthesis genes have shown expression in muscle tissue (Toledo 2019), crustacean lipid synthesis and oxidation primarily occurs in the hepatopancreas (the organ functionally equivalent to the liver and adipose tissue in vertebrates, and the fat body in insects) with lipids (predominantly phospholipids) being disseminated to other tissues, including muscle, via the hemolymph (O’Connor et al. 1968; Teshima et al. 1986; Garofalaki et al. 2006). A close examination of the hepatopancreas/hemolymph/muscle relationship could therefore inform attempts to ensure cultivated crustacean meat contains the correct lipid profiles. This might involve co-culturing cells or even a feeder cell system akin to Integriculture’s CultNet system, which allows media to be circulated between chambers containing different cell types (Hanyu and Kawashima 2017). However, because some lc-PUFAs, including EPA and DHA, are considered essential fatty acids in many crustaceans, unable to be synthesized by either organ (Zhao et al. 2015; Shu-Chien et al. 2017; Toledo 2019), such a system on its own is likely to be insufficient. As such, appropriate lipid profiles might be simply achieved with media or scaffold supplementation of just the essential—or perhaps all—required lipids that can be derived from plant sources or precision fermentation.

Alternatively, if driving endogenous adipogenesis is a consideration, then a deeper understanding of molecular mechanisms is needed. There is no known PPARγ equivalent in flies or crustaceans, although some suggest the multifunctional and molt-related nuclear receptor E75 may fill a similar role (Hong and Park 2010).

### Mollusks

The culture of molluscan cells is a relatively new and under-explored area, as reviewed by Yoshino et al. (2013).

#### Cephalopods

Cephalopod muscle structure is substantially different from amniotes and fish, with three sets of short (typically under one millimeter), mononucleated muscle fibers oriented in roughly perpendicular directions (Gosline and DeMont 1985; Kier 2016). Cephalopod fast fibers are distinguished by shorter sarcomeres and lower paramyosin content rather than by differences in myosin isoform expression as in vertebrates (Kier and Schachat 1992). As paramyosin contributes to the gel characteristics of squid meat (Sano et al. 1986), paramyosin content—along with muscle fiber ultrastructure—may be a key variable to optimize when developing differentiation protocols for cultivated cephalopod meat. The transcription factor NK4 is thought to play a role analogous to vertebrate Pax3/7 in determining myogenic precursors, while the Hh pathway is thought to be involved in muscle precursor proliferation (Zullo et al. 2017). For example, Grimaldi et al. found that Hh and Ptc are both expressed in cuttlefish myoblasts fated to become radial fast fibers and that inhibiting the pathway induced apoptosis and reduced the muscle precursors’ proliferation rate (Grimaldi et al. 2008). Myf5 and MyoD share functions between vertebrate and cephalopod lineages, while the factors involved in myotome determination and in differentiation are largely unknown (Zullo et al. 2017).

#### Bivalves

Our understanding of the molecular pathways involved in bivalve myogenesis is incomplete, though the Hh pathway has been implicated in myogenesis in oysters (Li et al. 2018). The insulin, PI3K-Akt, and mTOR pathways have been shown to correlate with growth rates in clams (Nie et al. 2021), though causal relationships have not been definitively established and effects in tissues besides muscle cannot be ruled out. In oysters, these pathways seem to be similarly correlated to muscle growth (Choi et al. 2018; Kim and Choi 2019) and a number of newly characterized insulin-like peptides appear to be critically, but variously, involved in muscle growth regulation (Li et al. 2021). A temporal expression analysis in scallop muscle has highlighted twist2 as a key component for myogenic differentiation of MSCs (Sun et al. 2021). In mussels, myogenic differentiation is influenced by the presence of particular extracellular matrix molecules, with collagen substrates tending to reversibly inhibit differentiation, and fibronectin, poly-L-lysine, and carbon-coated substrates promoting differentiation (Odintsova et al. 2010; Dyachuk 2013).

#### Gastropods

Myogenesis in gastropods is similarly poorly understood. Downregulation of abalone myostatin led to upregulation of the insulin pathway, which was taken as a proxy for somatic growth (Carrera-Naipil et al. 2016). Numerous microRNAs and long non-coding RNAs have been identified as differentially expressed between the muscle tissue of large and small abalone, suggesting that some of these might regulate myogenesis (Huang et al. 2018a, b). An abalone homolog of the homeobox gene *Mox* (also known as *Meox*) has been identified and, based on its expression pattern with the developing somite, was hypothesized to play a similar role in the development of the early mesoderm and the muscle lineage as in vertebrates (Hinman and Degnan 2002).

## Recommendations for Future Research

### Controlling Proliferation and Differentiation

While culture methods for fish embryonic and adult stem cells exist, optimizing culture media and growth conditions for long-term stemness maintenance and increased proliferation rates will help facilitate the scale-up process. This may be accomplished by optimizing component concentrations in existing formulations, likely assisted by statistical methods such as design of experiments (Cosenza et al. 2021) and testing recombinant species-specific growth factors (Venkatesan et al. 2022). Compounds such as IGF-2 (Rius-Francino et al. 2011) and anthocyanidins (Villasante et al. 2016), which have been demonstrated in fish myogenic stem cells to stimulate proliferation and increase expression of stem cell markers, respectively, may also be considered as media additives.

**Fig. 8:**
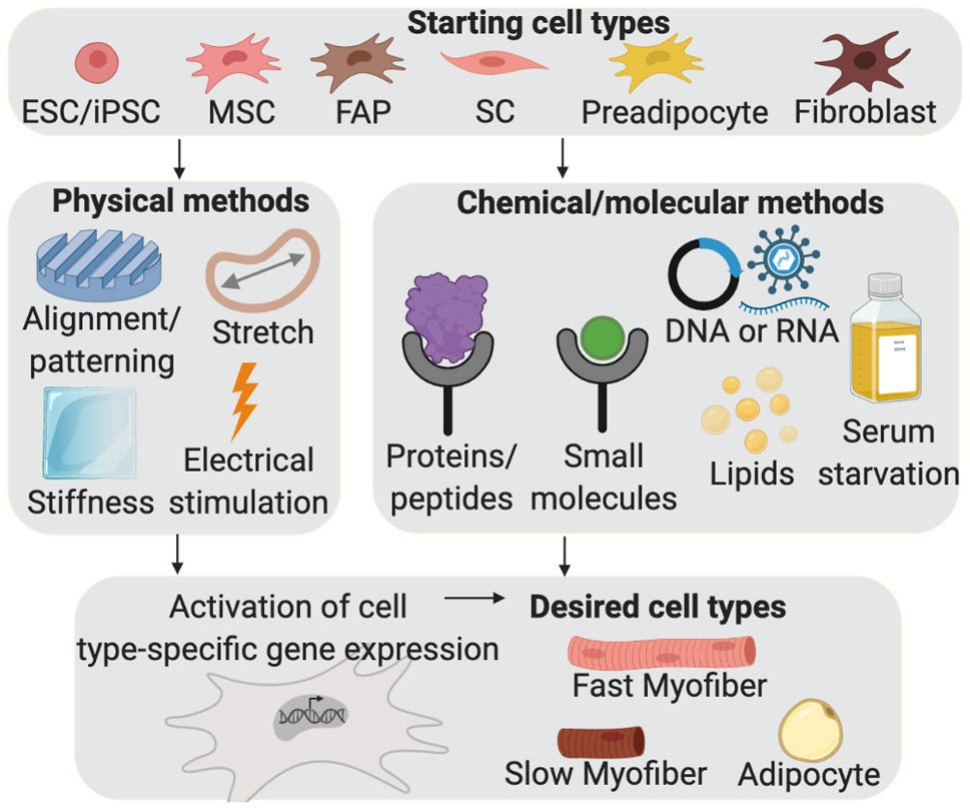
Several general strategies for inducing differentiation of cultured cells have been described in the literature and may be relevant to CM and CS, though not all have been applied to fish or other aquatic species. Starting and desired cell types shown are those especially relevant to CM and CS, but are not an exhaustive list. Strategies can be broadly categorized into physical methods and chemical or molecular methods, and multiple strategies may be combined to enhance differentiation

General strategies for inducing differentiation can be broadly divided into physical methods, in which the geometry or mechanical properties of the culture environment are manipulated, and chemical or molecular methods (Fig. 8). In both cases, gene expression patterns corresponding to the desired cell type are activated. Most of the existing data on fish myogenic and adipogenic differentiation come from studies in zebrafish. This makes zebrafish an invaluable tool for CS research (Potter et al. 2020), but also means that translating this knowledge to other species—a necessity if CS is to recreate the variety of seafood products available today—will require extensive research. Differences in the biology of marine and freshwater fish may make this challenge more difficult for marine species.

Physical methods have shown promising results in mammalian tissue engineering studies (Lee et al. 2022). They should be investigated as one possible strategy—perhaps in combination with chemical and molecular methods—for scalably inducing myogenic and adipogenic differentiation in cells from various seafood species. However, energy needs for strategies such as cyclic stretch and electrical stimulation must be considered.

Chemical and molecular methods have been better studied in fish cells, but more work is needed to ensure that the reagents used to control cell fate are food-safe, low-cost, scalable, and environmentally sustainable. It will be essential to consider the food safety implications of such reagents at the concentrations—including those of byproducts—expected in the final product (Ong et al. 2021). This includes perceived risks; for example, some consumers may be hesitant toward products in which genetic modification is used for cellular reprogramming or to induce differentiation. Such concerns may or may not correlate with scientifically backed safety concerns and may show regional variation (Bryant et al. 2020). Many of the compounds commonly used in differentiation media for research purposes, e.g., IBMX for adipogenesis, are not food-grade (Fish et al. 2020). Knowledge of the signaling pathways mediating the effects of such reagents on cell fate may inform efforts to replace them with safe and effective alternatives.

### Fish Myogenesis

Reagents previously demonstrated to induce myogenesis in fish stem cells include IGF1, IGF2, FGF2, GSK3b inhibitors, calpain inhibitors, and adenylate cyclase activators (see Table 2). Forskolin is a plant-based adenylate cyclase activator, sometimes taken as a dietary supplement, which has been shown to stimulate zebrafish SC proliferation and ESC-like cell myogenesis (Xu et al. 2013). While its safety as a supplement has not been conclusively demonstrated, existing evidence indirectly suggests the possibility of its safe use in inducing myogenesis in CS. Godard et al. (2005) used 25 mg forskolin per day to study weight loss in humans, while Xu et al. (2013) used 50 μM forskolin to induce zebrafish ESC-like cell myogenesis. Therefore, 1 l of the differentiation media would contain approximately the equivalent of one daily dose of forskolin. The amount remaining in a serving of CS—presumably considerably less since it is not known to accumulate in animal tissue—would need to be tested along with a rigorous safety profile.

Serum starvation is a reliable method for inducing myogenic differentiation in various species, including fish (Gabillard et al. 2010). While animal-derived serum is a poor choice for use in CM or CS, RNA sequencing of serum-starved cells was recently used to guide the development of a serum-free formulation for myogenesis of cultured bovine SCs (Messmer et al. 2022). Similar strategies could be employed for CS.

### Muscle Maturation, Fiber Type, and the Need for Consumer and Sensory Research

While myogenic differentiation is undoubtedly necessary for CS, it will be essential to understand the relationship between the extent of maturation and organoleptic properties. Given the differences in overall toughness and typical fiber lengths (Listrat et al. 2016), it would be reasonable to hypothesize that extensive fusion and maturation might be more necessary for terrestrial meat than fish.

Similarly, sensory and consumer research will determine how red muscle and skin—whether as part of the final product or removed after cooking and before eating—influence product acceptability. Where slow muscle is desirable, spatially defined cues that activate genes such as Mef2, Prdm1a, or the Hh pathway could be introduced. If cells develop into undesired slow fibers, strategies to activate class II Hdacs, Pbx, or Protein kinase A (PKA) may be helpful. However, because some evidence points to fast muscle as the “default” (Blagden et al. 1997; Du et al. 1997; Xu et al. 2000; Hinits and Hughes 2007), such cues may be unnecessary. In vivo, muscle pioneers are essential for maintaining the chevron shape of the myomeres, but make up a small percentage of slow fibers (Keenan and Currie 2019). It is conceivable that one might try to recapitulate the in vivo self-organization processes that lead to chevron formation (Rost et al. 2014; Tlili et al. 2019) using known signals for muscle pioneer or superficial slow fiber identity (Nguyen-Chi et al. 2012) as part of a CS bioprocess for whole cut filets. However, the muscle pioneers may otherwise be dispensable.

### Fish Adipogenesis

Development of food-grade differentiation protocols for fat will similarly require extensive optimization based on an understanding of the molecular pathways involved, followed by empirical testing. The fact that lipids tend to promote differentiation (Vegusdal et al. 2003) is an advantage since lipid-containing media may be used to simultaneously induce differentiation and control the lipid profile of the final product. However, tradeoffs may exist between the health benefits of omega-3 fats in the final product and their sometimes detrimental effects on differentiation (Huang et al. 2010; Liu et al. 2018).

### Invertebrates

Compared to fish, even less is known about differentiation into meat-relevant cell types in aquatic invertebrates. Muscle fiber structure and type composition vary considerably both from vertebrates and among invertebrates, and crustacean muscle structure varies considerably across the molt cycle (Mykles 1997), the organoleptic implications of which are not well understood. Knowledge of the molecular drivers and pathways involved in myogenesis and adipogenesis for these animals is particularly poor. However, as for fish, what is known can inform potential avenues for analysis.

Identification of a crustacean Nautilus homolog and its potential to initiate myogenesis in different stem cell types would be informative, as would investigating the effects of Twist, Mef2, and Pax3. There is still uncertainty around myostatin’s positive or negative effect on crustacean myogenesis, so this needs further elucidation. Some potential reagents to investigate are ecdysone and various growth factors, with emphasis on more species-relevant proteins.

As crustacean muscle tissue does not synthesize fat locally and lacks intramuscular adipocytes, fat supplementation via the media or scaffold may be necessary and sufficient to achieve appropriate fat profiles and distributions in cultivated crustacean meat.

Because crustaceans and insects share a close evolutionary relationship, knowledge about the mechanisms of myogenesis and adipogenesis may provide a “shortcut” to understanding these processes in crustaceans. It has also been suggested that cultured insect cells might be used directly as cell sources for cultivated CS, as the necessary culture conditions to grow such cells are both flexible and well-understood (Rubio et al. 2019b, c; Letcher et al. 2022). If the organoleptic properties of such products meet consumers’ expectations, this strategy may be effective at addressing the challenges related to the cost and scale of CS.

Research into mollusk myogenic differentiation could build on findings concerning molecular pathways such as Hh and various transcription factors. The work of Dyachuk (2013) and Odinstova et al. (2010) provide clear avenues to investigate culture substrate effects on myogenesis.

## Conclusion

The complexity of differentiation is a challenge for researchers attempting to identify the most efficient means of inducing the desired cellular behavior. It is also an opportunity because it offers many possible strategies. The task of future research will be to select those methods—or combinations of methods—that achieve the desired results in a manner that is cost-effective, food-safe, and sustainable.

For the potential benefits of CM and CS to be realized, it is essential that production costs be compatible with commodity meat prices. Several techno-economic assessments have been published, many of which model a wide range of scenarios and generate a similarly wide range of modeled costs. Published estimates include costs of USD $22—51/kg (Humbird 2021), $1.95—437,000/kg (Risner et al. 2021), $6.43—22,423/kg (Vergeer et al. 2021), $13.00—$30.40/kg (Negulescu et al. 2022), and $63/kg (Garrison et al. 2022). A primary cost driver—especially in nearer-term scenarios—is the culture medium, including macronutrients (primarily amino acids and glucose), growth factors, and other recombinant proteins (primarily albumin). Therefore, development of low-cost culture media is of paramount importance for CM and CS to achieve price parity with conventional animal products and be profitable. In some modeled scenarios where the cost of media had already been substantially reduced, capital expenditures and labor also made substantial contributions to the cost of production. Improvements to bioreactor technologies will help both to reduce capital expenditures and to increase the achievable cell densities and growth rates. When considering large-scale CM or CS production in bioreactors, many factors must be optimized to improve cell proliferation, including reducing shear-stress, adequately monitoring variables such as pH and carbon dioxide (Bellani et al. 2020), and removing waste metabolites from media. Promising solutions for the latter that are being pursued in for-profit organizations include medium recycling systems based on dialysis (Nahmias 2020) or genetic engineering techniques that reduce the production of toxic products such as ammonia (Genovese et al. 2021). Large-scale commercialization will also be enabled by innovations in standardized tissue sampling, cell banking, immortalization, reprogramming, scaffolding, and end product characterization. A recent analysis of spent media from cultured mouse and chicken cells revealed substantial variation in utilization of various key nutrients, suggesting that media formulations are unlikely to be interchangeable across species (O’Neill et al. 2022). It is likely to be generally true that cells will have different metabolic needs and different needs for specific growth factors to control their proliferation and differentiation depending on their species and cell type. By better understanding the needs of individual cell lines, it will be possible to improve the efficiency of CM and CS bioprocesses, thereby reducing both the environmental impacts and the cost of production for future products.

## Data Availability

Not applicable.
